# Current Trends in Volume Replacement Therapy and the Use of Synthetic Colloids in Small Animals—An Internet-Based Survey (2016)

**DOI:** 10.3389/fvets.2017.00140

**Published:** 2017-09-04

**Authors:** Ivayla D. Yozova, Judith Howard, Nadja E. Sigrist, Katja-Nicole Adamik

**Affiliations:** ^1^Institute of Veterinary, Animal and Biomedical Sciences, Massey University, Palmerston North, New Zealand; ^2^Diagnostic Clinical Laboratory, Vetsuisse Faculty, Department of Clinical Veterinary Medicine, University of Bern, Bern, Switzerland; ^3^Vetsuisse Faculty, Department of Small Animal Medicine, University of Zurich, Zurich, Switzerland; ^4^Emergency and Critical Care Section, Small Animal Clinic, Vetsuisse Faculty, Department of Clinical Veterinary Medicine, University of Bern, Bern, Switzerland

**Keywords:** dextran, fluid therapy, gelatin, hydroxyethyl starch, plasma expanders, synthetic colloids

## Abstract

The use of synthetic colloids (SCs), particularly hydroxyethyl starch (HES), in people has changed in recent years following new evidence raising concerns about their efficacy and safety. Although fluid therapy guidelines for small animals are often extrapolated from human medicine, little information exists on current practice in veterinary medicine. The objective of the present study was to investigate current fluid selection, use of plasma volume expanders including SCs, and recent changes in their use in small animal practice. An Internet-based survey was conducted, inviting veterinarians to report their practices in fluid resuscitation and colloid osmotic pressure support, their choice of SC, and perceived adverse effects and contraindications associated with SC use. There were 1,134 respondents from 42 countries, including 46% general practitioners and 38% diplomates. Isotonic crystalloids, HES, and hypertonic saline were chosen by most respondents for fluid resuscitation, and HES by 75% of respondents for colloid osmotic support. Dextran and gelatin were used by some European respondents. Human serum albumin was used more than canine albumin but 45% of respondents, particularly those from Australia and New Zealand, used no albumin product. The majority (70%) of respondents changed their practice regarding SCs in recent years (mostly by limiting their use), largely due to safety concerns. However, only 27% of respondents worked in an institution that had a general policy on SC use. Impaired renal function, coagulopathy, and hypertension were most often considered contraindications; impaired coagulation tests and increased respiratory rate were the most frequently perceived adverse effects. The use of HES remains widespread practice in small animals, regardless of geographic location. Nevertheless, awareness of safety issues and restrictions on the use of SCs imposed in human medicine seems to have prompted a decrease in use of SCs by veterinarians. Given the paucity of evidence regarding efficacy and safety, and differences in cohorts between human and veterinary critical care patients, studies are needed to establish evidence-based guidelines specific for dogs and cats.

## Introduction

Crystalloids and colloids have been widely used for plasma volume expansion in human and veterinary medicine for many decades (1–3). However, there is no general consensus in human and veterinary medicine as to the selection of fluid for plasma volume expansion despite numerous reviews and guidelines (4–9). Indeed, previous national and international surveys and observational studies found that fluid therapy practices are determined more by empirical preference than scientific evidence or physiological principles, with marked geographical and institutional disparity (1, 3, 10, 11). In recent years, randomized clinical trials (RCTs) and meta-analyses in human medicine have challenged previous concepts on the safety and efficacy of synthetic colloids (SCs), showing no benefit over crystalloids and, in some cases, linking the use of hydroxyethyl starch (HES) with acute kidney injury (AKI) and increased mortality (12–15). Although these findings have been disputed (16, 17), pharmacovigilance authorities have listed HES as contraindicated in septic and other critically ill patients, restricting its use in many countries (18, 19).

Recommendations for fluid resuscitation in small animals are often based on extrapolation from human medicine and veterinary experts’ opinions (2, 7, 9). As human critical care practice faced evidence questioning the benefit and restricting the use of SCs, small animal medicine is put to the challenge of scrutinizing its own practices with regards to fluid therapy. Merely adopting guidelines set forth in human medicine may not be wholly appropriate in small animals, given substantial differences in human and veterinary perioperative and intensive care unit (ICU) cohorts and the availability of species-specific blood products. Moreover, the use of SCs in constant rate infusions (CRIs) for colloid osmotic pressure (COP) support seems to be a practice unique to veterinary medicine, lacking corresponding data or trials in human medicine.

This paper presents the results of an international Internet-based survey evaluating current fluid therapy practices and factors influencing fluid selection in small animals. The survey was designed to question how fluids are used by veterinarians from different institutions and countries, the extent to which their practice may have changed over recent years, and examine the perceived risks and benefits associated with SC use.

## Materials and Methods

### Survey Composition and Characteristics

A commercial cloud-based survey development service (SurveyMonkey^®^, www.surveymonkey.com) was used to generate the questionnaire and gather participant responses. The question formats used were single- and multiple-answer multiple-choice, dichotomous questions, scaled and Likert-type ranking, and frequency scales with categorical or numerical ranking. All questions provided closed or combined closed/open answers whereby an “other” option provided a free-text field if none of the closed answers were valid. A total of 80 questions were developed, whereby contingency/filter questions were used to guide respondents to only those questions to which they were suited (Data Sheet S1 in Supplementary Material).

The survey was designed in two parts. An initial general part (Part 1) asked questions regarding respondent characteristics, qualifications, experience and area of practice, general use of fluids for resuscitation and COP support, and the preferred type of SC used. The second specific part (Part 2) asked questions about SC use, perceived adverse effects and contraindications, and participants’ recent changes in SC practices.

The study was approved by the Vetsuisse University IRB procedures. A preliminary survey was distributed to a pilot group (21 people) of in-house diplomates, residents, and interns. Questions were then amended for the final survey based on their feedback regarding question perspicuity and survey completion time.

### Data Collection and Analysis

A cover letter with the main investigator’s contact information, explaining the purpose of the survey, assuring confidentiality and encouraging recipients to invite their colleagues to participate was then distributed with a link to the survey to veterinary organizations (Data Sheet S2 in Supplementary Material). This was achieved by contacting organizations and requesting them to distribute the link to their members and/or post it on their homepages, and directly by email to members of organizations *via* LISTSERVs. In addition, some institutional heads of veterinary faculties were directly contacted with the request to distribute the survey within their institution and to their referring veterinarians (Data Sheet S3 in Supplementary Material). The survey was opened on 11th April 2016 and was closed on 14th May 2016.

All questions in Part 1 required an answer and only data from respondents answering all 17 questions were included for data analysis. For Part 2, the number of questions that required a response differed between respondents due to contingency questions. Responses to all questions in Part 2 were included.

## Results

### Results—Part 1

A total of 1,134 respondents completed at least Part 1 of the survey (Data Sheet S4 in Supplementary Material).

#### Respondent Characteristics

**Figure 1:**
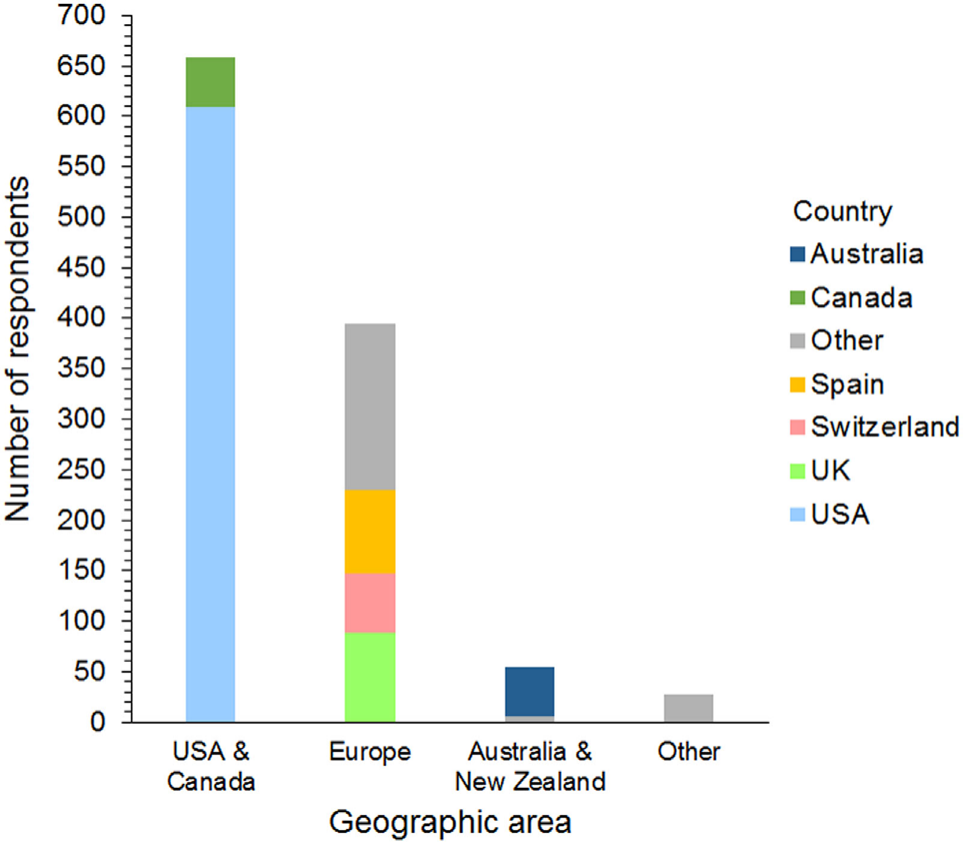
Frequency chart showing the geographic distribution of the 1,134 survey respondents. Countries represented by 30 or more respondents are shown separately within each geographic area.

**Figure 2:**
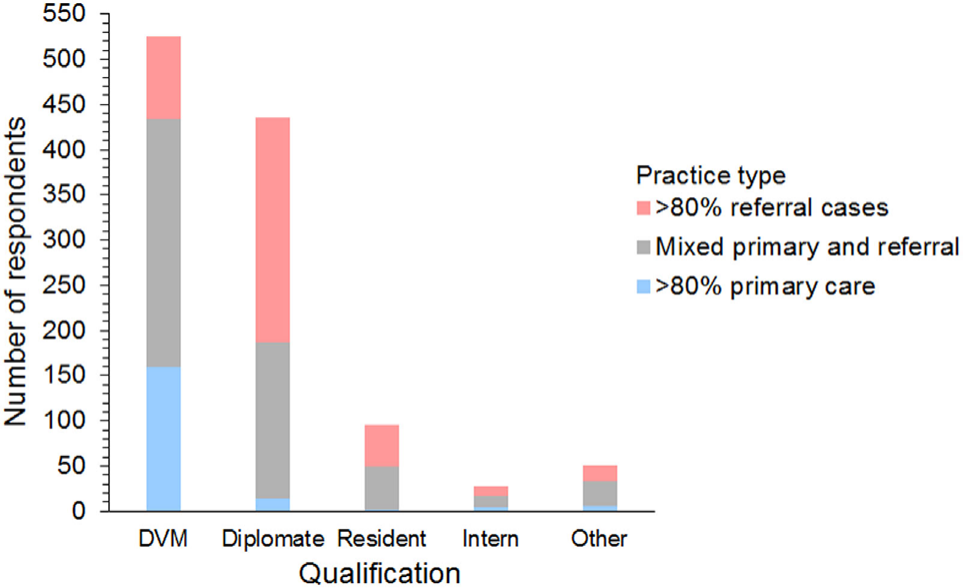
Frequency chart showing the qualifications and case types seen by the 1,134 survey respondents.

Respondents were 394 men and 740 women from 42 different countries. These included 658 (58%) from the USA and Canada, 395 (35%) from Europe, 54 (5%) from Australia and New Zealand, and 27 (2%) from 17 other countries (Figure 1). The workplace setting of respondents was 489 (43%) in private practice, 319 (28%) in a university hospital, and 326 (29%) in a specialty or other type of practice. These included primary care, referral, and mixed primary and referral practices (Figure 2). The number of years’ experience of the respondents was ≤5 years in 237 (21%), 6–10 years in 322 (28%), 11–15 years in 233 (21%), 16–20 years in 126 (11%), and >20 years in 216 (19%) of respondents.

Respondents included 525 (46%) general practitioners, 435 (38%) board-certified diplomates, 95 (8%) residents, 28 (3%) interns, and 51 (5%) others/unspecified. Diplomates included 216 (45%) emergency and critical care specialists (ACVECC and ECVECC), 90 (19%) internists (ACVIM and ECVIM), 84 (17%) anesthetists (ACVAA and ECVAA), 75 (16%) surgeons (ACVS and ECVS), 6 (1%) neurologists (ECVN), and 15 (3%) unspecified board-certified specialists.

#### Intravenous Fluid Resuscitation

**Figure 3:**
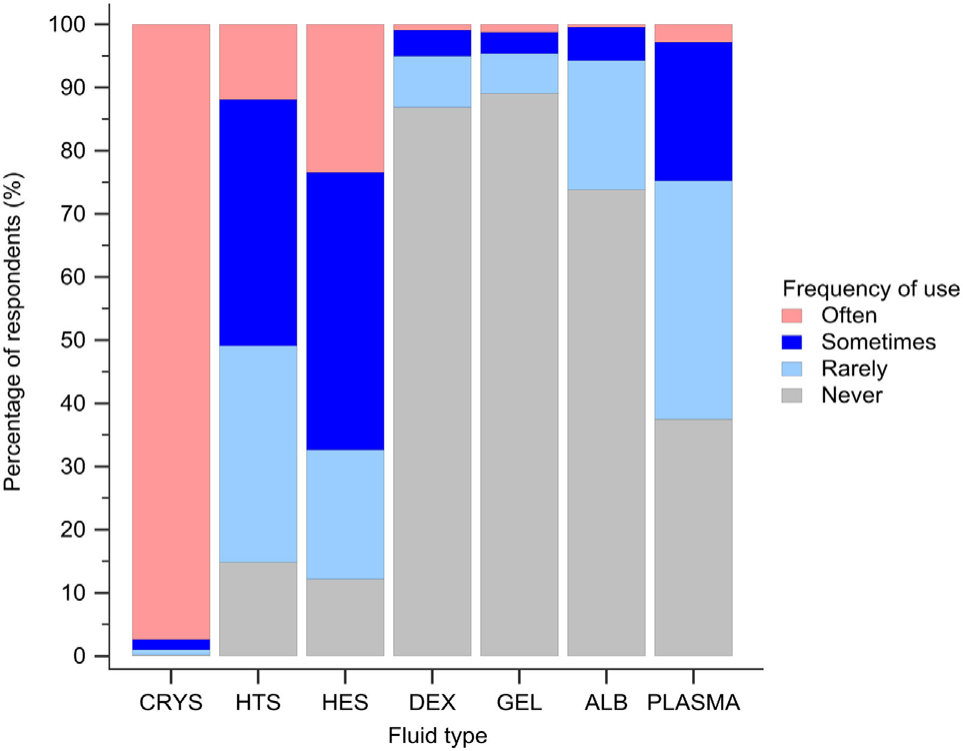
Frequency chart showing the relative frequency with which the 1,134 survey respondents use intravenous solutions for fluid resuscitation. CRYS, crystalloids; HTS, hypertonic saline; HES, hydroxyethyl starch; DEX, dextran; GEL, gelatin; ALB, albumin.

**Figure 4:**
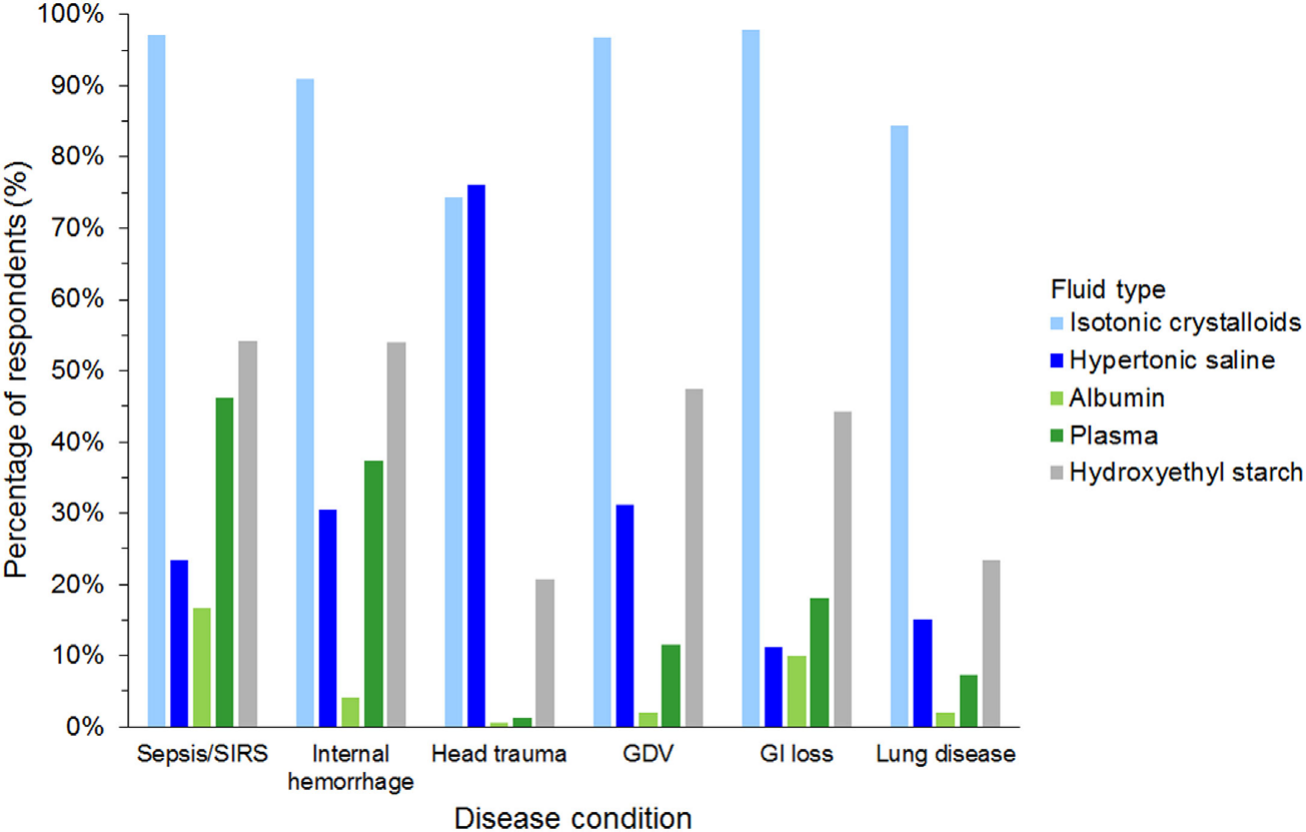
Frequency chart showing the type of intravenous solutions used by the 1,134 survey respondents for fluid resuscitation in specific disease conditions. SIRS, systemic inflammatory response syndrome; GDV, gastric dilatation-volvulus; GI loss, gastrointestinal loss.

Isotonic crystalloids, HES, and hypertonic saline were reported to be used sometimes or often for fluid resuscitation by 1,123 (99%), 730 (64%), and 577 (51%) of respondents, respectively (Figure 3). Gelatin, dextran, and albumin were reported to be never used by 1,010 (89%), 985 (87%), and 837 (74%) of respondents, respectively (Figure 3). Solutions used by over 50% of respondents with specific disease conditions were isotonic crystalloids and HES in sepsis/SIRS and internal hemorrhage, isotonic crystalloids, and hypertonic saline in head trauma, and isotonic crystalloids in gastric dilatation-volvulus, gastrointestinal fluid loss, and lung disease (Figure 4).

#### Intravenous Colloid Osmotic Support

**Figure 5:**
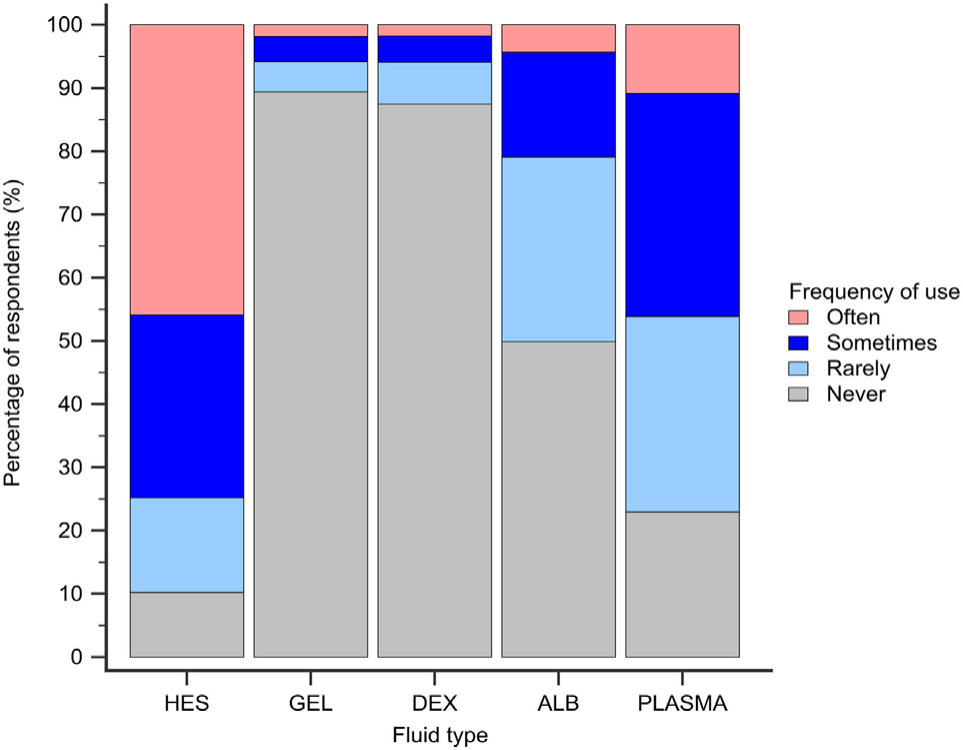
Frequency chart showing the relative frequency with which the 1,134 survey respondents use intravenous solutions for colloid osmotic support. HES, hydroxyethyl starch; GEL, gelatin; DEX, dextran; ALB, albumin.

**Figure 6:**
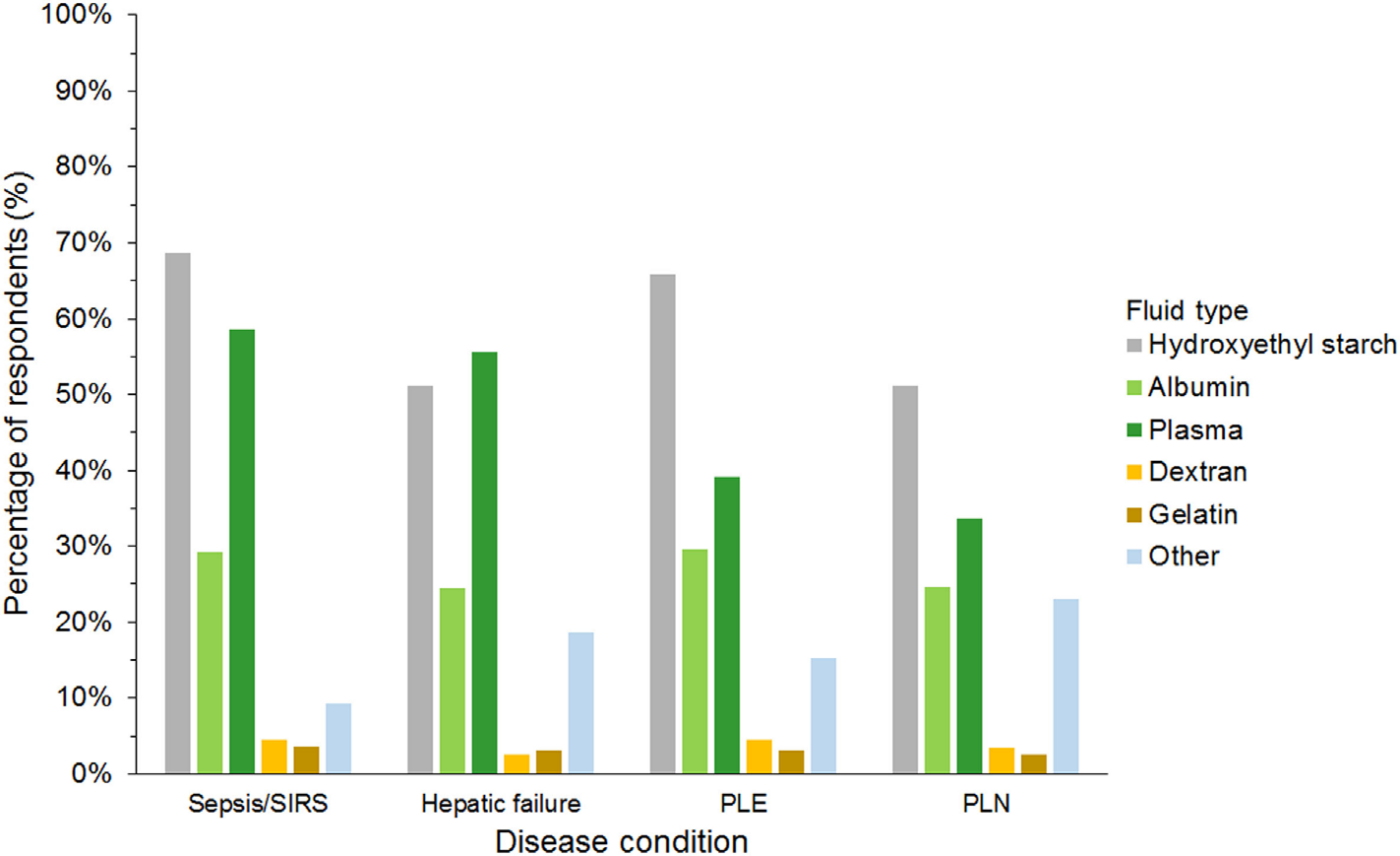
Frequency chart showing the type of intravenous solutions used by the 1,134 survey respondents for colloid osmotic support in specific disease conditions. SIRS, systemic inflammatory response syndrome; PLE, protein-losing enteropathy; PLN, protein-losing nephropathy.

Hydroxyethyl starch and plasma were reported to be used sometimes or often for COP support by 848 (75%) and 523 (46%) of respondents, respectively (Figure 5). Gelatin, dextran, and albumin were reported to be never used by 1,014 (89%), 992 (87%), and 566 (50%) of respondents, respectively. Solutions used by over 50% of respondents with specific disease conditions were HES and plasma in sepsis/SIRS and hepatic failure, and HES in protein-losing enteropathy and nephropathy (Figure 6).

#### Use of Albumin Preparations

Five and 20–25% human serum albumin was used by 262 (23%) and 321 (28%) of respondents, respectively, and canine albumin was used by 182 (16%) of respondents, whereby some respondents used more than one albumin product. No albumin product was used by 505 (45%) of respondents, including 89% of respondents from Australia and New Zealand. Canine albumin was used by 21% of respondents from the USA and Canada but was seldom used in other geographic regions (Data Sheet S5 in Supplementary Material).

#### Choice of SC

**Figure 7:**
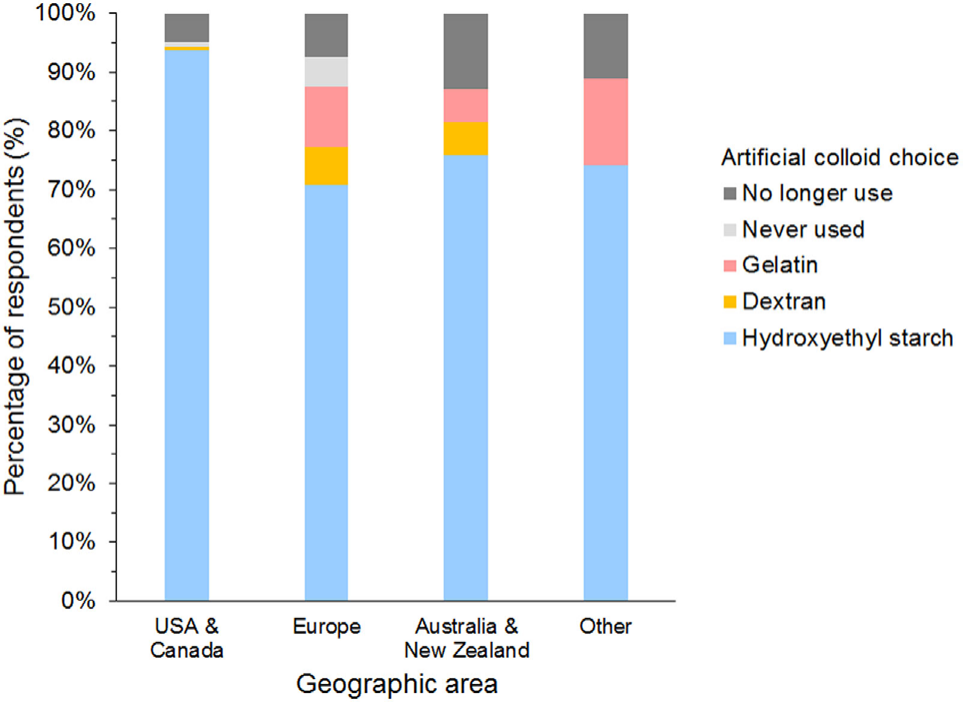
Frequency chart showing the primary synthetic colloid used by the 1,134 survey respondents from different geographic areas.

The most frequently used SC was HES in 958 (85%), gelatin in 49 (4%), and dextran in 31 (3%) of respondents (Figure 7). A further 71 (6%) of respondents reported having discontinued using SCs, and 25 (2%) had never used SCs. Of those that had discontinued SC use, 65/71 (92%) previously used HES.

#### Institutional Policies on the Use of SCs

Only 304 (27%) reported that a general policy or guideline for the use of SCs existed in their workplace. Most respondents (726, 64%) reported no general policy and a further 104 (9%) did not know if one existed (Data Sheet S6 in Supplementary Material).

### Results—Part 2

Of the 1,134 respondents who completed the general part of the survey, 1,051 also responded to questions in the second specific part (Part 2).

#### Use of SCs

**Figure 8:**
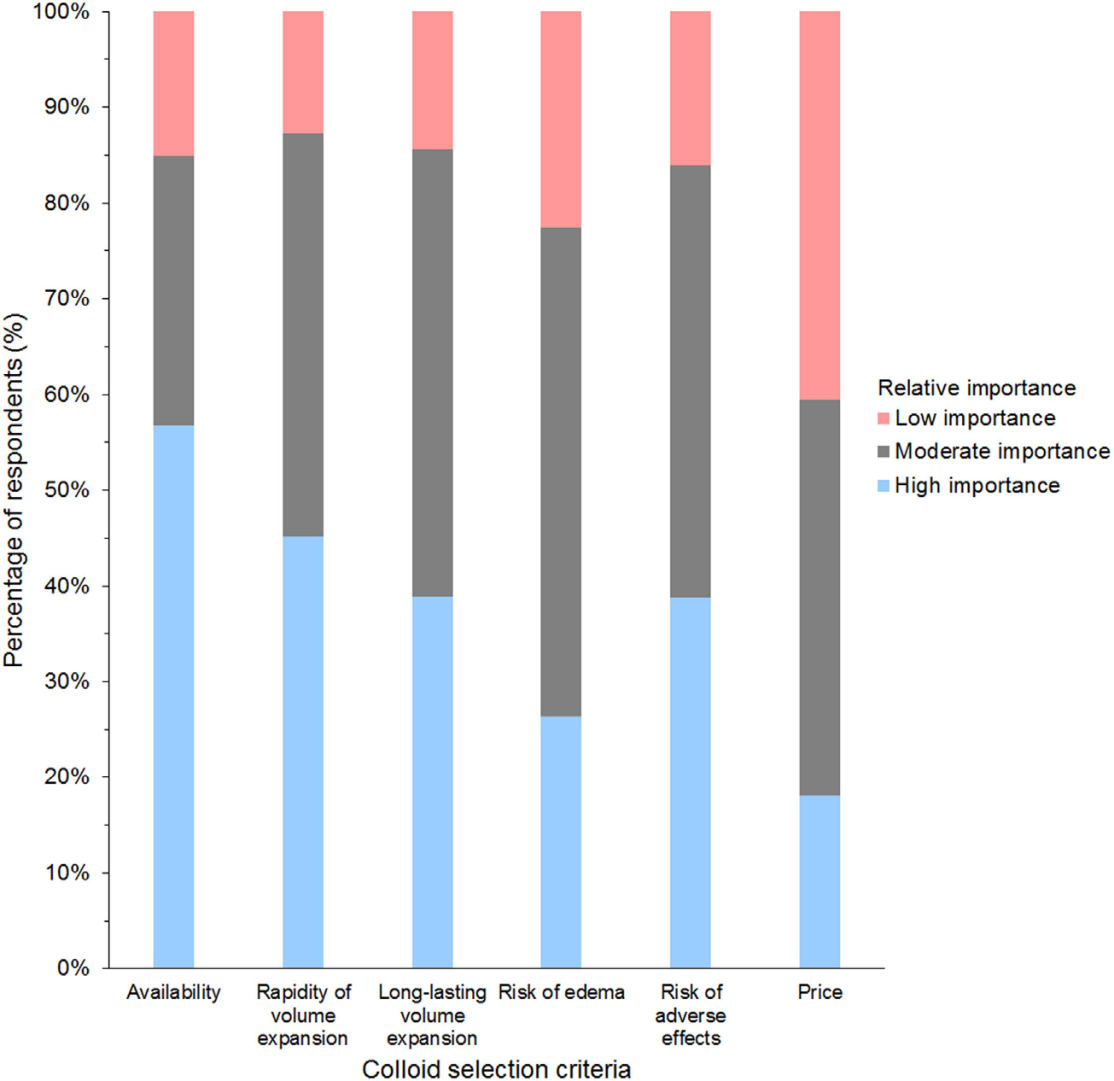
Frequency chart showing the relative importance of criteria reported as considerations in the selection of synthetic colloids by 1,024 survey respondents.

Of 947 respondents using HES, 64% used tetrastarch, 25% hetastarch, 8% pentastarch, and 3% did not specify HES type (Data Sheet S7 in Supplementary Material). Use of dextran and gelatin was generally limited to respondents in Europe, whereby gelatin was the main SC in 24% of respondents from the UK and dextran in 15% of respondents from Spain. The criterion reported most often as having high importance in influencing the choice of SC was availability (58% of respondents); the criterion reported most often as having low importance was price (42% of respondents) (Figure 8).

The bolus dose of SCs reported for fluid resuscitation varied somewhat but was generally lower in cats than dogs (Table 1). Likewise, the maximum daily dose was generally lower in cats than in dogs (Table 2; Data Sheet S8 in Supplementary Material). For tetrastarch, the most frequently reported maximum daily doses were 11–20 and 21–30 ml/kg/day for dogs (in 46 and 27% of tetrastarch users, respectively), and ≤10 and 11–20 ml/kg/day for cats (in 26 and 47% of tetrastarch users, respectively). Similarly, for hetastarch, the most frequently reported maximum daily doses were 11–20 and 21–30 ml/kg/day for dogs (in 58 and 18% of hetastarch users, respectively), and ≤10 and 11–20 ml/kg/day for cats (in 35 and 45% of hetastarch users, respectively).

**Table 1:** Bolus dose of synthetic colloids used for fluid resuscitation by survey respondents.

ml/kg	Hydroxyethyl starch users *N* (%)		Gelatin users *N* (%)		Dextran users *N* (%)	
	Dogs	Cats	Dogs	Cats	Dogs	Cats
≤5	**417 (45%)**	**747 (81%)**	11 (24%)	**31 (69%)**	5 (20%)	**11 (44%)**
6–10	**367 (40%)**	97 (11%)	**25 (56%)**	7 (16%)	**8 (32%)**	4 (16%)
11–15	58 (6%)	26 (3%)	2 (4%)	1 (2%)	4 (16%)	1 (4%)
16–20	60 (7%)	8 (1%)	3 (7%)	1 (2%)	4 (16%)	2 (8%)
>20	5 (1%)	3 (< 1%)	0 (0%)	0 (0%)	1 (4%)	0 (0%)
I don’t know/not applicable	20 (2%)	46 (5%)	4 (9%)	5 (11%)	3 (12%)	**7 (28%)**

*Frequently reported doses (>25% of respondents) are given in bold font*.

**Table 2:** Maximum daily dose of synthetic colloids used for fluid resuscitation by survey respondents.

ml/kg/day	Hydroxyethyl starch users^a^ *N* (%)		Gelatin users *N* (%)		Dextran users*N* (%)	
	Dogs	Cats	Dogs	Cats	Dogs	Cats
≤10	59 (6%)	**269 (29%)**	4 (9%)	**13 (29%)**	2 (8%)	6 (24%)
11–20	**460 (50%)**	**424 (46%)**	**16 (36%)**	**14 (31%)**	**8 (32%)**	8 (32%)
21–30	217 (23%)	115 (12%)	**14 (31%)**	6 (13%)	5 (20%)	1 (4%)
31–40	70 (8%)	18 (2%)	1 (2%)	1 (2%)	2 (8%)	1 (4%)
41–50	65 (7%)	23 (3%)	2 (4%)	1 (2%)	1 (4%)	0 (0%)
>50	6 (<1%)	3 (<1%)	1 (2%)	1 (2%)	1 (4%)	0 (0%)
I don’t know/not applicable	50 (5%)	75 (8%)	7 (16%)	9 (20%)	6 (24%)	**9 (36%)**

*Frequently reported doses (>25% of respondents) are given in bold font*.

*^a^For details of the maximum daily dose for each HES product, see supplemental material*.

#### CRI of SCs

**Figure 9:**
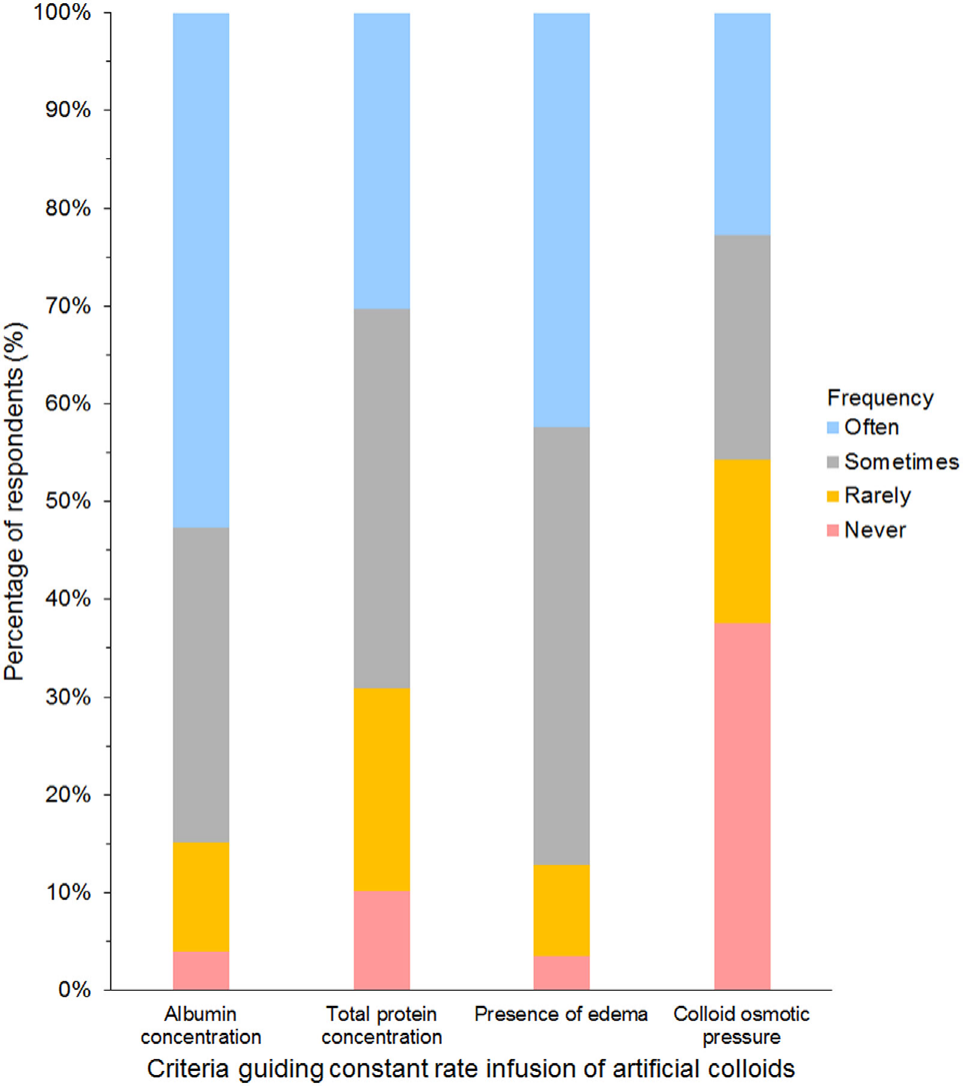
Frequency chart showing the relative importance of criteria used to guide the decision to use synthetic colloids as a constant rate infusion reported by 681 survey respondents.

Criteria most frequently reported to be used sometimes or often to guide the decision to use SCs in CRI were albumin concentration (85%) and presence of edema (87%, Figure 9). The criterion most frequently reported to be used rarely or never was COP (54%). The CRI dose of SCs reported by the respondents is presented in Table 3. A slight majority of respondents reported that they limited the duration of CRI to a maximum of 3 days (Table 4). However, 25% of respondents reported no maximum time limit.

**Table 3:** Constant rate infusion dose of synthetic colloids used by survey respondents.

ml/kg/h	Hydroxyethyl starch users *N* (%)		Gelatin users *N* (%)		Dextran users*N* (%)	
	Dogs	Cats	Dogs	Cats	Dogs	Cats
≤1.0	**308 (48%)**	**432 (67%)**	**9 (39%)**	**12 (57%)**	**5 (45%)**	**9 (82%)**
1.1–2.0	**250 (39%)**	120 (19%)	**10 (43%)**	3 (14%)	**4 (36%)**	1 (9%)
2.1–3.0	28 (4%)	14 (2%)	0 (0%)	0 (0%)	1 (9%)	0 (0%)
>3.0	15 (2%)	5 (<1%)	1 (4%)	1 (5%)	0 (0%)	0 (0%)
I don’t know/not applicable	46 (7%)	76 (12%)	3 (13%)	5 (22%)	1 (9%)	1 (9%)

*Frequently reported doses (>25% of respondents) are given in bold font*.

**Table 4:** Maximum limit for the duration of constant rate infusions of synthetic colloids by survey respondents.

Limit	Respondents (*N*)			
	Hydroxyethyl starch	Dextran	Gelatin	All
1 day	131	2	8	141 (21%)
3 days	332	7	13	352 (52%)
1 week	18	1	0	19 (3%)
No limit	166	1	2	169 (25%)

#### Changes in SC Use

Of the 65 respondents who had stopped using HES, 59 (91%) had stopped during the past 5 years. The most frequent reason given was concerns regarding its safety (Table 5). Most respondents who had stopped using HES reported observing neither an increase nor a decrease in the incidence of edema, hypotension, length of hospitalization, or mortality in animals they treated. Although 39% reported using vasopressors more frequently, 51% reported no increase in their use of vasopressors since stopping their use of HES. Products used instead of HES by respondents who had stopped using HES were isotonic crystalloids in 55 (85%), plasma in 41 (63%), hypertonic saline in 37 (57%), albumin in 18 (28%), and other/unspecified in 2 (3%) of respondents.

**Table 5:** Reasons reported by survey respondents for stopping or changing their use of hydroxyethyl starch (HES).

Reasons given	Respondents *N* (%)		
	Stopped using HES (*N* = 65)	Changed using HES (*N* = 650)	All (*N* = 715)
Concerns regarding its safety	59 (91%)	536 (82%)	595 (83%)
Concerns regarding its efficacy	38 (59%)	300 (46%)	338 (47%)
Availability issues	12 (19%)	158 (24%)	170 (24%)
Other reasons	10 (15%)	18 (3%)	28 (4%)

**Figure 10:**
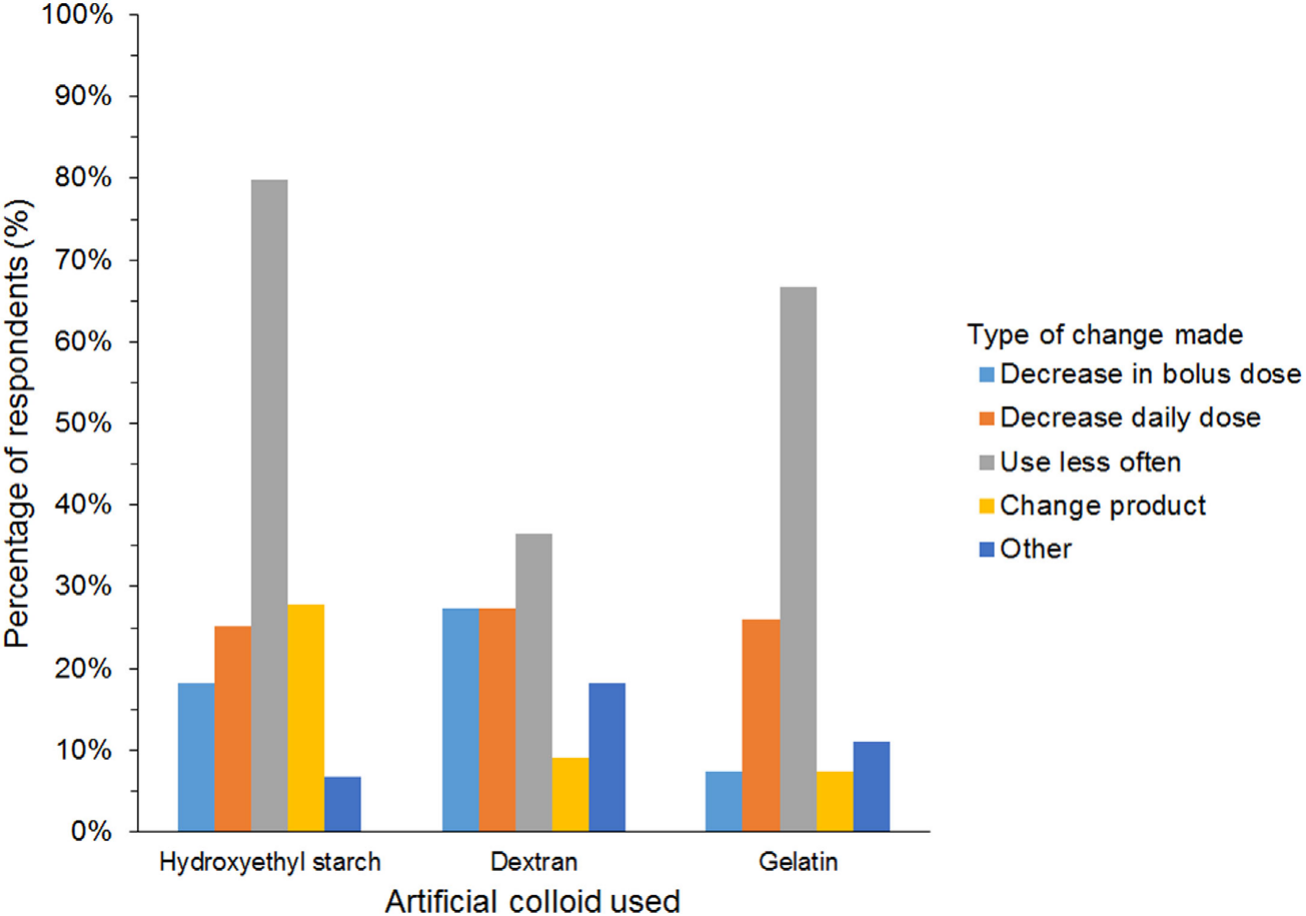
Frequency chart showing the changes in synthetic colloid use over the past 5 years and/or because of new recommendations reported by survey respondents (650 hydroxyethyl starch users; 27 gelatin users; 11 dextran users).

**Figure 11:**
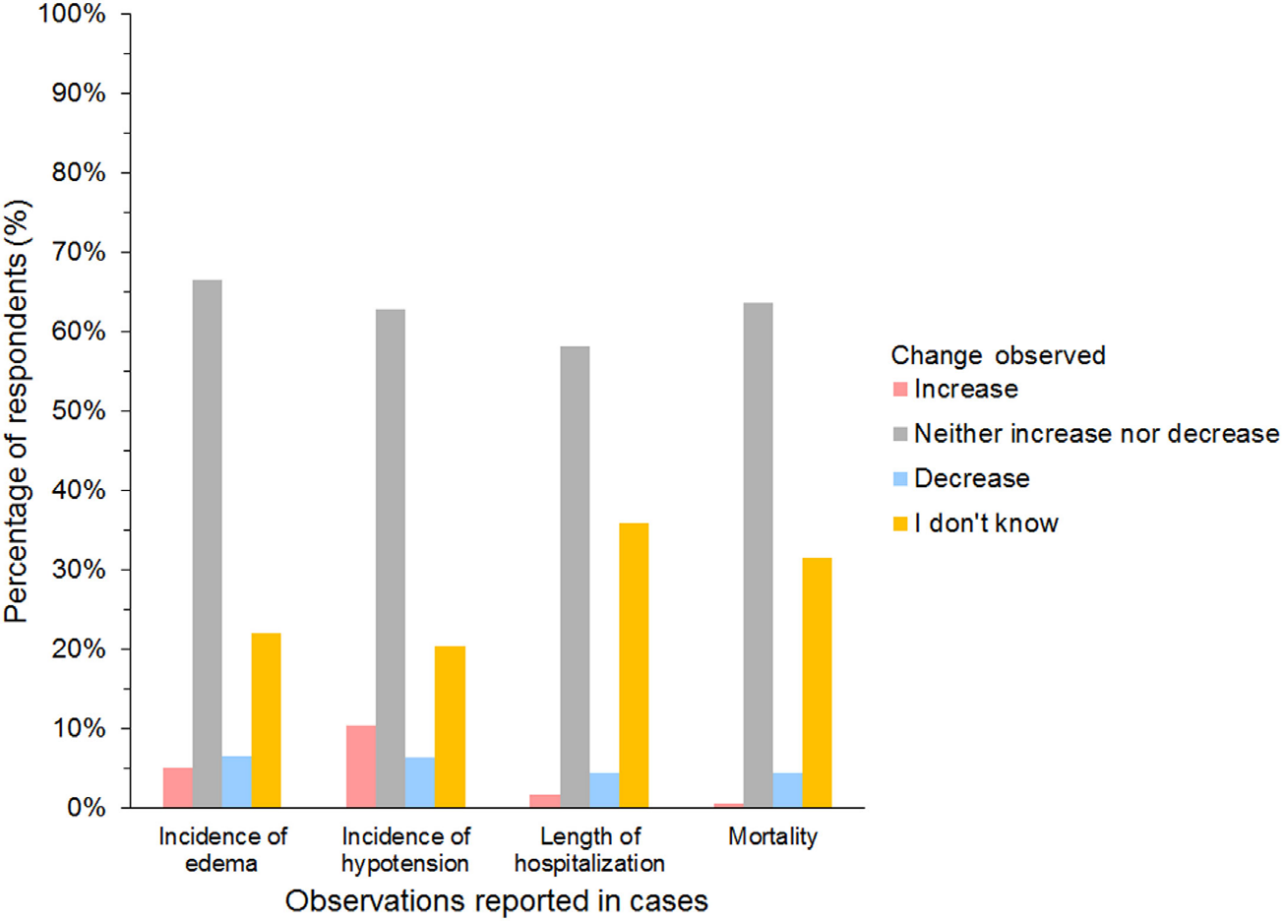
Frequency chart showing incidence of adverse reactions noted by survey respondents since changing their use of synthetic colloids (654 hydroxyethyl starch users; 27 gelatin users; 12 dextran users).

A further 654/927 (71%) of HES users agreed that they had changed their use of HES over the last 5 years but continued using HES. The most frequent reason was concerns regarding its safety (Table 5). In addition, 27/45 (60%) of gelatin users and 12/25 (48%) of dextran users reported changing their use of SCs. The most frequent change in SC use reported by respondents using all three SCs was less frequent use (Figure 10). Products used instead of HES by respondents that had changed their use of HES were isotonic crystalloids in 364 (57%), hypertonic saline in 254 (40%), plasma in 248 (39%), albumin in 116 (18%), and other/unspecified in 28 (4%) of respondents. Most reported neither increase nor decrease in the incidence of edema or hypotension, the length of hospitalization, or mortality since changing their use of SCs (Figure 11).

#### Adverse Reactions Associated with SCs

Of 927 HES users, 25 dextran users and 45 gelatin users answering the question, no specific adverse reaction associated with SC use was reported by more than 30% of respondents (Data Sheet S9 in Supplementary Material). The most frequently reported adverse reaction was increased respiratory rate/effort (29% of HES users; 22% of gelatin users; 32% of dextran users) and impaired coagulation tests for HES users (29% of respondents).

#### Perceived Contraindications for the Use of SCs

**Figure 12:**
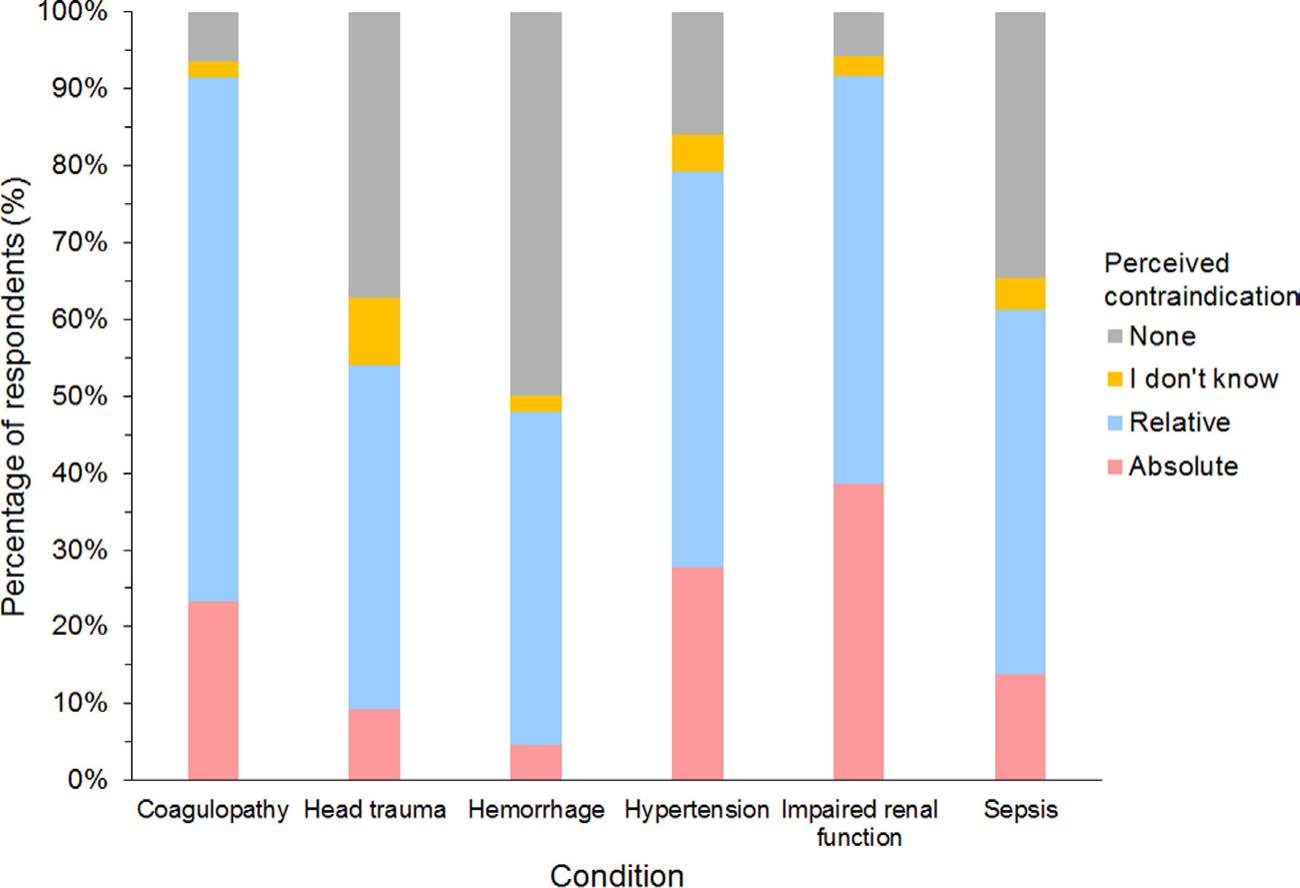
Frequency chart showing relative and absolute contraindications for the use of hydroxyethyl starch perceived by 632 survey respondents.

Of 632 HES users responding to the question, impaired renal function and coagulopathy were the two most common conditions considered either relative or absolute contraindications for using HES, and more than a quarter of respondents considered impaired renal function and hypertension as absolute contraindications (Figure 12). However, no condition was considered an absolute contraindication by most respondents. Of the 27 gelatin users responding, 85% considered coagulopathy, hypertension, and impaired renal function as relative or absolute contraindications, but only hypertension was considered an absolute contraindication by more than a quarter of respondents (26%). Only 10 dextran users responded to the question. Here again, hypertension was considered a relative or absolute contraindication by 9/10 and an absolute contraindication by 5/10 respondents.

## Discussion

This is the first major international survey on the use of SCs in small animals. Respondents represented a varied group of veterinarians with diverse qualifications and experience, working in different clinical settings. However, the majority (58%) of respondents were from North America, which likely created some degree of bias. As respondents from USA and Canada almost never used gelatin and dextran and represented 64% of HES users, survey results may be distorted toward practices particular to this geographic area. This must be borne in mind when interpreting findings of this survey. Furthermore, gelatin and dextran users were very low in numbers, and results given as percentages of these users were affected by responses from few individuals.

Based on this survey, the most common resuscitation fluids used are isotonic crystalloids followed by HES. Similarly, isotonic crystalloids are preferred to SCs for fluid resuscitation in human ICUs (3, 10, 11), and a recent survey examining fluid therapy practices in dogs and cats found isotonic crystalloids were more commonly used than colloids (20).

Despite widespread use of HES by respondents from all countries, and gelatin and dextran in Europe, most respondents declared having no guideline or policy on the use of SCs in their workplace. However, our findings suggest that guidelines may be more common in Europe and some other geographic areas than in North America, possibly reflecting differences in new legislation for SC use in people or differences in attitudes toward the freedom of veterinarians to treat their cases as they see fit. Nevertheless, a small number of veterinarians have stopped using SCs and a large majority have changed their SC practices in recent years (mostly by using them less frequently). These changes were largely due to concerns regarding safety and, to a lesser extent, efficacy. Although evidence investigating the safety of SCs in small animals is growing, most published clinical studies on mortality and SC-related AKI are non-randomized and retrospective studies on HES. One retrospective cohort study in dogs showed a significantly higher incidence of AKI in dogs treated with 10% pentastarch (6%, *n* = 11) compared to dogs not receiving HES (2%, *n* = 4) (21). Conversely, four retrospective studies (in dogs and cats) did not find a significant difference in blood creatinine concentrations and AKI grades between critically ill animals treated with tetrastarch (6% HES-130/0.4) and those not receiving HES (22–25). However, one of these studies did find an association between the number of days of tetrastarch administration and an increase in AKI grade (24). In contrast, a CRI of 6% tetrastarch (50 ml/kg/day) in healthy dogs for 3 days was not found to impact renal function or cause lesions consistent with osmotic nephrosis (26). Nevertheless, most of these veterinary studies lacked statistical power due to small sample size, and further research is necessary to elucidate the real scope and severity of HES-related kidney injury in small animals. Only rare reports on renal side effects of gelatins and dextrans in small animals have been published (27). Recently, in a canine hemorrhagic shock model comparing dogs treated with 6% tetrastarch, 4% gelatin, fresh whole blood, and crystalloids, AKI was observed in all dogs regardless of treatment, with some evidence suggesting greater kidney injury in gelatin-treated dogs (28).

Although safety concerns lead most respondents to alter their use of SCs, more respondents reported availability to be of high importance in their choice of SC than risk of adverse effects. This apparent discrepancy in the relative importance given to safety issues likely reflects differences in opinions between those who did change their practice and all other SC users responding to the survey. Given the relative paucity of veterinary literature on HES safety, changes in SC practices are likely due largely to respondents’ awareness of recent human studies and new guidelines in people. In addition, in some countries, such as the UK, HES solutions were recalled from the market following the European Medicines Agency alert in 2013 (29). In consequence, some HES users may have been obliged to discontinue the use of HES or consider using other SCs.

Despite concerns regarding safety, a minority of respondents reported experiencing adverse effects of SCs in their cases. This apparent discrepancy between human and veterinary medicine is likely the result of major differences in cohorts and treatment complexity. Furthermore, HES-related human RCTs were often criticized for their poor quality and more recently for refusal to share raw data (16, 17, 30). Of those respondents changing their SC practice in the present survey, few observed changes in the incidence of edema, hypotension, length of hospitalization, or mortality in their cases. However, some 39% of former HES users did declare an increase use of vasopressors. The fluid most frequently used instead of HES by respondents stopping or changing their use of HES was isotonic crystalloids. This is similar to current changes in fluid resuscitation practices in people reported in a recent international cross-sectional study (31).

Although these findings together suggest a disparity between perceived and reported risks associated with SCs, it is unlikely that many respondents logged such events in their cases. Moreover, increased respiratory rate/effort, impaired coagulation tests, and hypertension were indeed observed by users of all three SCs. Previous studies have reported impaired coagulation tests associated with SC use in both people and small animals (32, 33). However, despite the increasing body of literature assessing coagulation impairment (33–37), no association with clinical bleeding has thus far been documented in dogs or cats, but this was noted as an observed adverse reaction by some respondents in the present survey. Increased respiratory rate/effort and hypertension may be explained by volume overload leading to transient hypertension and increased hydrostatic pressure leading to pulmonary edema. Another explanation would be an allergic reaction although this has not been reported in animals to the authors’ knowledge. Gelatin and dextran are reported to have the most allergic potential in people (38), and both were occasionally associated with allergic reactions by survey respondents.

Coagulopathy and impaired renal function were most often considered contraindications for the use of HES although increased serum creatinine was rarely reported to be an observed adverse effect. This is in accordance with recent studies demonstrating a transient impairment of canine platelet function and whole blood coagulation (33–37) but no effect of HES administration on serum creatinine or urinary biomarkers (22–25, 27). Although sepsis, severe coagulopathy, intracranial hemorrhage, and impaired renal function are now considered absolute contraindications in many countries for the use of HES in people (18, 19), these were mostly considered only relative contraindications by respondents in this survey. Surprisingly, many respondents considered hypertension as either a relative or absolute contraindication although hypertension is not noted in HES product labeling, which specified only volume overload as a contraindication (39).

Although dosages of SCs varied, these were largely similar between respondents. The most commonly used HES preparation was tetrastarch, but the most frequent maximal daily dose used was ≤20 ml/kg/day. This may be due to previous use of hetastarch, for which the recommended doses in people were not exceeding 20 ml/kg/day (40). Furthermore, the only currently available veterinary HES product (6% HES 130/0.4 in 0.9% sodium chloride solution) has a maximal daily dose of 20 ml/kg (41). Whether this dose is based on extrapolation from older HES generations remains unclear. Interestingly, recommended doses for tetrastarch for people appear to vary significantly (between 30 and 50 ml/kg/day) in different countries (39, 42, 43).

Doses used for gelatin were similar to those used for HES although daily limits for gelatin use are neither recommended by manufacturers nor reported elsewhere to the authors’ knowledge (44, 45). In general, survey respondents used lower bolus doses of SCs in dogs and cats compared to those recommended in people (2, 7). While lower doses may be reasonable in cats, the reason for this tendency in dogs is unclear.

The preferred HES carrier solution (buffered electrolyte-balanced versus saline) was not investigated in this survey, but may be important in the selection of HES products. Indeed, several human trials showed an association between saline-based colloids and acid–base and electrolytes disturbances, including hyperchloremia, and decreased base excess, bicarbonate and anion gap (46).

Most survey respondents use SCs in CRIs, a practice unique to veterinary medicine. Continuous rate infusion doses were within the currently recommended ranges of 20–30 ml/kg/day (7). Based on the present survey, CRIs of SCs are widely used for COP support in dogs and cats. As COP depends on the number of osmotically active particles, low molecular weight preparations, such as HES 130/0.4, may be expected to exert greater COP at similar concentrations compared to products of high molecular weight (2). However, a recent study in dogs with hypoalbuminemia showed that although plasma COP was maintained, it did not increase following an initial dose of 5 ml/kg 6% HES 130/0.4 over 6h, followed by CRI of 24 ml/kg/day (47).

A limitation on the duration of SC CRIs was variably reported by respondents in this survey. This may reflect both varied knowledge about the effects of cumulative doses and tissue storage in humans as well as the lack of published facts about such effects in dogs and cats (48).

Even though RCTs failed to show benefits of albumin infusions in human ICU patients (49, 50), albumin is still widely recommended and used in people (31, 51). In the present survey, just over one half of respondents used albumin preparations, with human serum albumin most frequently used, despite potential adverse effects reported in dogs (52). Respondents from Australia and New Zealand rarely used albumin products and canine albumin was mostly used in the USA and Canada, suggesting marked geographic disparity in the use of albumin products. One reason for this is that lyophilized canine albumin is currently only distributed in USA, Canada, Hong Kong, Singapore, and Taiwan.

Data from this survey suggest that, regardless of their geographic location or qualifications, veterinarians continue using HES and other SCs. Nevertheless, and despite a lack of practice guidelines, a notable proportion of respondents have limited their use of SCs based on an awareness of potential deleterious effects. As little data exist in veterinary medicine, changes in SC practice uncovered in this survey are likely due largely to results of human RCTs. Given differences in cohorts between veterinary and human ICU patients, studies are needed to establish evidence-based guidelines for the use of SCs in dogs and cats.

## Author Contributions

IY helped design the survey questionnaire, interpreted data, and wrote the manuscript. JH designed the survey questionnaire, interpreted data, and revised the manuscript. NS helped design the survey questionnaire and revised the manuscript. KNA designed the study questionnaire, interpreted data, and revised the manuscript. All authors read and approved the final manuscript.

## Conflict of Interest Statement

The authors declare that the research was conducted in the absence of any commercial or financial relationships that could be construed as a potential conflict of interest.

## Abbreviations

AKI, acute kidney injury; COP, colloid osmotic pressure; CRI, constant rate infusion; HES, hydroxyethyl starch; ICU, intensive care unit; RCT, randomized clinical trial; SC, synthetic colloid.
