# Supplementary material for: Current Trends in Volume Replacement Therapy and the Use of Synthetic Colloids in Small Animals—An Internet-Based Survey (2016)
Source: Front Vet Sci. 2017 Sep 4;4:140. doi: 10.3389/fvets.2017.00140 (PMC5591339; doi:10.3389/fvets.2017.00140)
Supplement: Supplementary file 1 [file Data_Sheet_1.PDF]

1.

Thank you for agreeing to take part in this important survey assessing current trends in volume replacement therapy and the use of colloids in small animal practice.

Completing this survey should take only about 6-8 minutes and your answers are collected anonymously.

If you have any comments or questions regarding this survey, please contact me by e-mail: katja.adamik@vetsuisse.unibe.ch.

Katja-Nicole Adamik Dip. ACVECC, Dip. ECVECC
Emergency and Critical Care Service
Small Animal Clinic
Vetsuisse Faculty
University of Bern
Switzerland

This survey has been reviewed by the Vetsuisse University IRB procedures for research.

2. General information - 1

* 1. I work in:

- ◯ Private practice
- ◯ University hospital
- ◯ Specialty practice/other

If specialty/other, please specify:

* 2. The total number of veterinarians working in my clinic/practice is:

- ◯ 1-10
- ◯ 11-20
- ◯ 21-30
- ◯ 31-40
- ◯ >40

* 3. The cases I see are:

- ◯ Mostly (>80%) referral cases
- ◯ Mostly (>80%) primary care cases
- ◯ A mixture of referral and primary care cases

* 4. I am a:

- ◯ Intern
- ◯ Resident
- ◯ Diplomate
- ◯ DVM / practioner
- ◯ Other

If other, please specify:

3. Resident

* 5. My board program is:

- [ ] ACVAA
- [ ] ACVECC
- [ ] ACVIM
- [ ] ACVS
- [ ] ECVAA
- [ ] ECVECC
- [ ] ECVIM
- [ ] ECVS
- [ ] Other

If other, please specify:

4. Diplomate

* 6. My board certification is:

- [ ] ACVAA
- [ ] ACVECC
- [ ] ACVIM
- [ ] ACVS
- [ ] ECVAA
- [ ] ECVECC
- [ ] ECVIM
- [ ] ECVS
- [ ] Other

If other, please specify:

5. General information - 2

* 7. I am:

- Male
- Female

* 8. I have practiced veterinary medicine for:

- 0-5 years
- 6-10 years
- 11-15 years
- 16-20 years
- >20 years

* 9. The country in which I practice is:

6. General Fluid Resuscitation -1

* 10. I use the following solutions for **fluid resuscitation/shock** (select one answer per line):

	Never	Rarely	Sometimes	Often
Isotonic crystalloids	○	○	○	○
Hypertonic saline	○	○	○	○
Hydroxyethyl starch	○	○	○	○
Gelatin	○	○	○	○
Dextran	○	○	○	○
Albumin	○	○	○	○
Plasma	○	○	○	○

7. General Fluid Resuscitation -2

* 11. I use the following solutions for **fluid resuscitation/shock** in the **following conditions** (select all that apply):

	Isotonic crystalloids	Hypertonic saline	Hydroxyethyl starch	Gelatin	Dextran	Albumin	Plasma	Other/not applicable
Sepsis/SIRS	☐	☐	☐	☐	☐	☐	☐	☐
Internal hemorrhage	☐	☐	☐	☐	☐	☐	☐	☐
Head trauma	☐	☐	☐	☐	☐	☐	☐	☐
Gastric dilatation volvulus	☐	☐	☐	☐	☐	☐	☐	☐
Gastrointestinal fluid losses	☐	☐	☐	☐	☐	☐	☐	☐
Severe lung disease	☐	☐	☐	☐	☐	☐	☐	☐

8. Colloids General - 1

* 12. I use the following solutions for general **colloid osmotic pressure support** (select one answer per line):

	Never	Rarely	Sometimes	Often
Hydroxyethyl starch	○	○	○	○
Gelatin	○	○	○	○
Dextran	○	○	○	○
Albumin	○	○	○	○
Plasma	○	○	○	○

9. Colloids General - 2

* 13. I use the following solutions for **colloid osmotic pressure support** in the **following conditions** (select all that apply):

	Hydroxyethyl starch	Gelatin	Dextran	Albumin	Plasma	Other/not applicable
Sepsis/SIRS	☐	☐	☐	☐	☐	☐
Protein-losing nephropathy	☐	☐	☐	☐	☐	☐
Protein-losing enteropathy	☐	☐	☐	☐	☐	☐
Hepatic failure	☐	☐	☐	☐	☐	☐

10. Colloids General - 3

* 14. I use the following **albumin** preparation(s) (select all that apply):

- [ ] Human serum albumin 5%
- [ ] Human serum albumin 20% or 25%
- [ ] Lyophilized canine albumin
- [ ] Other
- [ ] I don't use any

If other, please specify:

* 15. My practice has a general policy/guideline on the use of colloids:

- ◯ Agree
- ◯ Disagree
- ◯ I don't know

11. Transfer to colloid choice

* 16. The synthetic colloid I **most frequently use** is:

- ○ Hydroxyethyl starch
- ○ Gelatin
- ○ Dextran
- ○ I no longer use any
- ○ I have never used any

12. Colloid I mostly use

* 17. The synthetic colloid I **mostly used** was:

- ◯ Hydroxyethyl starch
- ◯ Gelatin
- ◯ Dextran

13. I stopped HES - 1

* 18. I have stopped using hydroxyethyl starch (HES) since:

- ◯ 0-5 years
- ◯ 6-10 years
- ◯ 11-15 years
- ◯ 16-20 years
- ◯ >20 years

* 19. I stopped using HES because of (select all that apply):

- ☐ Availability issues
- ☐ Concerns regarding its safety
- ☐ Concerns regarding its efficacy
- ☐ Other reasons

If other reasons, please specify:

14. I stopped HES - 2

* 20. Since I stopped using HES, I have observed the following in my cases (select one answer per line):

	Increase	Decrease	Neither	I don't know
Incidence of edema	○	○	○	○
Incidence of hypotension	○	○	○	○
Mortality	○	○	○	○
Length of hospitalization	○	○	○	○

* 21. The product(s) I now use instead of HES is/are (select all that apply):

- ☐ Hypertonic saline
- ☐ Isotonic crystalloids
- ☐ Plasma
- ☐ Albumin
- ☐ Other

If other, please specify:

* 22. Since I stopped using HES, I use more vasopressors:

- ○ Agree
- ○ Disagree
- ○ I don't know

15. I stopped Gelatin - 1

* 23. I have stopped using gelatin since:

- ○ 0-5 years
- ○ 6-10 years
- ○ 11-15 years
- ○ 16-20 years
- ○ >20 years

* 24. I stopped using gelatin because of (select all that apply):

- ☐ Availability issues
- ☐ Concerns regarding its safety
- ☐ Concerns regarding its efficacy
- ☐ Other reasons

If other reasons, please specify:

16. I stopped gelatin - 2

* 25. Since I stopped using gelatin, I have observed the following in my cases (select one answer per line):

	Increase	Decrease	Neither	I don't know
Incidence of edema	◯	◯	◯	◯
Incidence of hypotension	◯	◯	◯	◯
Mortality	◯	◯	◯	◯
Length of hospitalization	◯	◯	◯	◯

* 26. The product(s) I now use instead of gelatin is/are (select all that apply):

- ☐ Hypertonic saline
- ☐ Isotonic crystalloids
- ☐ Plasma
- ☐ Albumin
- ☐ Other

If other, please specify:

* 27. Since I stopped using gelatin, I use more vasopressors:

- ◯ Agree
- ◯ Disagree
- ◯ I don't know

17. I stopped dextran - 1

* 28. I have stopped using dextran since:

- ○ 0-5 years
- ○ 6-10 years
- ○ 11-15 years
- ○ 16-20 years
- ○ >20 years

* 29. I stopped using dextran because of (select all that apply):

- ☐ Availability issues
- ☐ Concerns regarding its safety
- ☐ Concerns regarding its proven efficacy
- ☐ Other reasons.

If other reasons, please specify:

18. stopped dextran - 2

* 30. Since I stopped using dextran, I have observed the following in my cases (select one answer per line):

	Increase	Decrease	Neither	I don't know
Incidence of edema	○	○	○	○
Incidence of hypotension	○	○	○	○
Mortality	○	○	○	○
Length of hospitalization	○	○	○	○

* 31. The product(s) I now use instead of dextran is/are (select all that apply):

- ☐ Hypertonic saline
- ☐ Isotonic crystalloids
- ☐ Plasma
- ☐ Albumin
- ☐ Other

If other, please specify:

* 32. Since I stopped using dextran, I use more vasopressors:

- ○ Agree
- ○ Disagree
- ○ I don't know

19. HES fluid resuscitation - 1

* 33. The hydroxyethyl starch (HES) product I mostly use is:

* 34. My choice of synthetic colloid is based on considerations of (select one answer per line):

	Little importance	Moderate importance	High importance
Rapidity of volume expansion	○	○	○
Long-lasting volume expansion	○	○	○
Risk of interstitial edema	○	○	○
Risk of adverse effects	○	○	○
Availability	○	○	○
Price	○	○	○

20. HES fluid resuscitation - 2

* 35. The **bolus dose (ml/kg)** of HES I use for fluid **resuscitation/shock** is about (select one answer per line):

	I don't know/ not applicable	0-5	6-10	11-15	16-20	>20
**Dogs**:	◯	◯	◯	◯	◯	◯
**Cats**:	◯	◯	◯	◯	◯	◯

* 36. The **maximum daily dose (ml/kg/24h)** of HES I use is about (select one answer per line):

	I don't know/ not applicable	0-10	11-20	21-30	31-40	41-50	>50
**Dogs**:	◯	◯	◯	◯	◯	◯	◯
**Cats**:	◯	◯	◯	◯	◯	◯	◯

* 37. I have observed the following associated with HES administration (select one answer per line):

	Agree	Disagree	I don't know
Hypertension	◯	◯	◯
Bleeding	◯	◯	◯
Impaired coagulation tests	◯	◯	◯
Increased respiratory rate/effort	◯	◯	◯
Increased serum creatinine	◯	◯	◯
Allergic reactions/anaphylaxis	◯	◯	◯

* 38. I have changed how I use HES over the last 5 years and/or because of new recommendations:

◯ Agree

◯ Disagree

21. Tranfer HES CRI yes/no

* 39. I use HES as a **constant rate infusion** for colloid osmotic pressure support:

- Agree
- Disagree

22. did change practice HES -1

* 40. I changed my use of HES because of (select all that apply):

- [ ] Availability issues
- [ ] Concerns regarding its safety
- [ ] Concerns regarding its proven efficacy
- [ ] Other

If other, please specify:

* 41. The main change(s) I made is/are (select all that apply):

- [ ] I decreased the daily dose
- [ ] I decreased the bolus dose
- [ ] I use it less often
- [ ] I changed from one HES product to another
- [ ] Other

If other, please specify:

23. did change practice HES -2

* 42. Since changing how I use HES, I have observed the following in my cases (select one answer per line):

	Increase	Decrease	Neither	I don't know
Incidence of edema	○	○	○	○
Incidence of hypotension	○	○	○	○
Mortality	○	○	○	○
Length of hospitalization	○	○	○	○

* 43. Product(s) I use **more frequently since** changing how I use HES is/are (select all that apply):

- ☐ Hypertonic saline
- ☐ Isotonic crystalloids
- ☐ Plasma
- ☐ Albumin
- ☐ Other
- ☐ None

If other, please specify:

[ ]

24. did change practice HES -3

* 44. I consider the following conditions **contraindications** for using HES (select one answer per line):

	No contraindication	Relative contraindication	Absolute contraindication	I don't know
Impaired renal function	○	○	○	○
Head trauma	○	○	○	○
Coagulopathy	○	○	○	○
Hemorrhage	○	○	○	○
Hypertension	○	○	○	○
Sepsis	○	○	○	○

* 45. I use HES as a **constant rate infusion** for colloid osmotic pressure support:

○ Agree

○ Disagree

25. Gelatins fluid resuscitation -1

* 46. The gelatin product I mostly use is:

* 47. My choice of synthetic colloid is based on considerations of (select one answer per line):

	Little importance	Moderate importance	High importance
Rapidity of volume expansion	◯	◯	◯
Long-lasting volume expansion	◯	◯	◯
Risk of interstitial edema	◯	◯	◯
Risk of adverse effects	◯	◯	◯
Availability	◯	◯	◯
Price	◯	◯	◯

26. Gelatins fluid resuscitation - 2

* 48. The **bolus dose (ml/kg)** of gelatin I use for **fluid resuscitation/shock** is about (select one answer per line):

	I don't know/ not applicable	0-5	6-10	11-15	16-20	>20
**Dogs**:	○	○	○	○	○	○
**Cats**:	○	○	○	○	○	○

* 49. The **maximum daily dose (ml/kg/24h)** of gelatin I use is about (select one answer per line):

	I don't know/ not applicable	0-10	11-20	21-30	31-40	41-50	>50
**Dogs**	○	○	○	○	○	○	○
**Cats**	○	○	○	○	○	○	○

* 50. I have observed the following associated with gelatin administration (select one answer per line):

	Agree	Disagree	I don't know
Hypertension	○	○	○
Bleeding	○	○	○
Impaired coagulation tests	○	○	○
Increased respiratory rate/effort	○	○	○
Increased serum creatinine	○	○	○
Allergic reactions/anaphylaxis	○	○	○

* 51. I have changed how I use gelatin over the last 5 years and/or because of new recommendations:

○ Agree

○ Disagree

27. Transfer Gelatin CRI yes/no

* 52. I use gelatin as a **constant rate infusion** for colloid osmotic pressure support:

- ◯ Agree
- ◯ Disagree

28. did change practice gelatin -1

* 53. I changed my use of gelatin because of (select all that apply):

- [ ] Availability issues
- [ ] Concerns regarding its safety
- [ ] Concerns regarding its proven efficacy
- [ ] Other

If other, please specify:

* 54. The main change(s) I made is/are (select all that apply):

- [ ] I decreased the daily dose
- [ ] I decreased the bolus dose
- [ ] I use it less often
- [ ] I changed from one gelatin product to another
- [ ] Other

Of other, please specify:

29. did change practice gelatin -2

* 55. Since changing how I use gelatin, I have observed the following in my cases (select one answer per line):

	Increase	Decrease	Neither	I don't know
Incidence of edema	○	○	○	○
Incidence of hypotension	○	○	○	○
Mortality	○	○	○	○
Length of hospitalization	○	○	○	○

56. Product(s) I use **more frequently since changing** how I use gelatin is/are (select all that apply):

- ○ Hypertonic saline
- ○ Isotonic crystalloids
- ○ Plasma
- ○ Albumin
- ○ Other
- ○ None

If other, please specify

[ ]

30. did change practice gelatin -3

* 57. I consider the following conditions **contraindications** for using gelatin (select one answer per line):

	No contraindication	Relative contraindication	Absolute contraindication	I don't know
Impaired renal function	◯	◯	◯	◯
Head trauma	◯	◯	◯	◯
Coagulopathy	◯	◯	◯	◯
Hemorrhage	◯	◯	◯	◯
Hypertension	◯	◯	◯	◯
Sepsis	◯	◯	◯	◯

* 58. I use gelatin as a **constant rate infusion** for colloid osmotic pressure support:

◯ Agree

◯ Disagree

31. Dextran fluid resuscitation - 1

* 59. The dextran product I mostly use is:

* 60. My choice of synthetic colloid is based on considerations of (select one answer per line):

	Little importance	Moderate importance	High importance
Rapidity of volume expansion	○	○	○
Long-lasting volume expansion	○	○	○
Risk of interstitial edema	○	○	○
Risk of adverse effects	○	○	○
Availability	○	○	○
Price	○	○	○

32. Dextran fluid resuscitation - 2

* 61. The **bolus dose (ml/kg)** of dextran I use for **fluid resuscitation/shock** is about (select one answer per line):

	I don't know/ not applicable	0-5	6-10	11-15	16-20	>20
**Dog**:	◯	◯	◯	◯	◯	◯
**Cat**:	◯	◯	◯	◯	◯	◯

* 62. The **maximum daily dose (ml/kg/24h)** of dextran I use is about (select one answer per line):

	I don't know/not applicable	0-10	11-20	21-30	31-40	41-50	>50
**Dogs**	◯	◯	◯	◯	◯	◯	◯
**Cats**	◯	◯	◯	◯	◯	◯	◯

* 63. I have observed the following associated with dextran administration (select one answer per line):

	Agree	Disagree	I don't know
Hypertension	◯	◯	◯
Bleeding	◯	◯	◯
Impaired coagulation tests	◯	◯	◯
Increased respiratory rate/effort	◯	◯	◯
Increased serum creatinine	◯	◯	◯
Allergic reactions/anaphylaxis	◯	◯	◯

* 64. I have changed how I use dextran over the last 5 years and/or because of new recommendations:

◯ Agree

◯ Disagree

33. Transfer Dextran CRI yes/no

* 65. I use dextran as a **constant rate infusion** for colloid osmotic pressure support:

◯ Agree

◯ Disagree

34. did change practice dextran -1

* 66. I changed my use of dextran because of (select all that apply):

- [ ] Availability issues
- [ ] Concerns regarding its safety
- [ ] Concerns regarding its efficacy
- [ ] Other

If other, please specify

____

* 67. The main change(s) I made is/are (select all that apply):

- [ ] I decreased the daily dose
- [ ] I decreased the bolus dose
- [ ] I use it less often
- [ ] I changed from one gelatin product to another
- [ ] Other

If other, please specify:

____

35. did change practice dextran -2

* 68. Since changing how I use dextran, I have observed the following in my cases (select one answer per line):

	Increase	Decrease	Neither	I don't know
Incidence of edema	◯	◯	◯	◯
Incidence of hypotension	◯	◯	◯	◯
Mortality	◯	◯	◯	◯
Length of hospitalization	◯	◯	◯	◯

* 69. Product(s) I use **more frequently since changing** how I use dextran is/are (select all that apply):

◯ Hypertonic saline

◯ Isotonic crystalloids

◯ Plasma

◯ Albumin

◯ Other

◯ None

If other, please specify:

[ ]

36. did change practice dextran -3

* 70. I consider the following conditions **contraindications** for using dextran (select one answer per line):

	No contraindication	Relative contraindication	Absolute contraindication	I don't know
Impaired renal function	○	○	○	○
Head trauma	○	○	○	○
Coagulopathy	○	○	○	○
Hemorrhage	○	○	○	○
Hypertension	○	○	○	○
Sepsis	○	○	○	○

* 71. I use dextran as a **constant rate infusion** for colloid osmotic pressure support:

○ Agree

○ Disagree

37. CRI HES

* 72. The following guide my decision to use **HES** as a **constant rate infusion (CRI)** (select one answer per line):

	Never	Rarely	Sometimes	Often
Serum albumin concentration	○	○	○	○
Serum total protein concentration	○	○	○	○
Presence of peripheral edema	○	○	○	○
Colloid osmotic pressure	○	○	○	○

* 73. I use a HES **CRI dose (ml/kg/h)** of about (select one answer per line):

	I don't know/ not applicable	0-1.0	1.1-2.0	2.1-3.0	>3.0
Dogs	○	○	○	○	○
Cats	○	○	○	○	○

* 74. I generally limit the **maximum total duration** of HES CRI to about:

- ○ 1 day
- ○ 3 days
- ○ 1 week
- ○ No limit

38. CRI gelatin

* 75. The following guide my decision to use gelatin as a **constant rate infusion (CRI)** (select one answer per line):

	Never	Rarely	Sometimes	Often
Serum albumin concentration	◯	◯	◯	◯
Serum total protein concentration	◯	◯	◯	◯
Presence of peripheral edema	◯	◯	◯	◯
Colloid osmotic pressure	◯	◯	◯	◯

* 76. I use a gelatin **CRI dose (ml/kg/h)** of about (select one answer per line):

	I don't know/ not applicable	0-1.0	1.1-2.0	2.1-3.0	> 3.0
Dogs	◯	◯	◯	◯	◯
Cats	◯	◯	◯	◯	◯

* 77. I generally limit the **maximum total duration** of gelatin CRI to about:

◯ 1 day

◯ 3 days

◯ 1 week

◯ No limit

39. CRI dextran

* 78. The following guide my decision to use **dextran** as a **constant rate infusion (CRI)** (select one answer per line):

	Never	Rarely	Sometimes	Often
Serum albumin concentration	◯	◯	◯	◯
Serum total protein concentration	◯	◯	◯	◯
Presence of peripheral edema	◯	◯	◯	◯
Colloid osmotic pressure	◯	◯	◯	◯

* 79. I use a dextran **CRI dose (ml/kg/h)** of about (select one answer per line):

	I don't know/ not applicable	0-1.0	1.1-2.0	2.1-3.0	>3.0
Dogs	◯	◯	◯	◯	◯
Cats	◯	◯	◯	◯	◯

* 80. I generally limit the **maximum total duration** of dextran CRI to about:

◯ 1 day

◯ 3 days

◯ 1 week

◯ No limit

40. Last page - Thank you

Thank you for taking part in this survey.

If you have any comments or questions regarding this survey, please contact me by e-mail: katja.adamik@vetsuisse.unibe.ch.

Katja-Nicole Adamik Dip. ACVECC, Dip. ECVECC
Emergency and Critical Care Service
Small Animal Clinic
Vetsuisse Faculty
University of Bern
Switzerland
