# Supplementary material for: Current Trends in Volume Replacement Therapy and the Use of Synthetic Colloids in Small Animals—An Internet-Based Survey (2016)
Source: Front Vet Sci. 2017 Sep 4;4:140. doi: 10.3389/fvets.2017.00140 (PMC5591339; doi:10.3389/fvets.2017.00140)
Supplement: Supplementary file 2 [file Data_Sheet_S2_to_S9.PDF]

Supplemental data S2 to S9

Data S2

E-Mail text to veterinarians/ veterinary organizations:

Dear XXX
We are interested in assessing current trends in volume replacement therapy and the use of colloids in small animals. Please support us by completing a short survey and feel free to forward this e-mail to your colleagues.

Survey Link: https://www.research.net/r/VetSColloids
Please follow the link to the survey. All responses are collected anonymously and treated confidentially.

Expected Completion Time: 6-8 minutes
Survey Close Date: 9th May 2016 (was prolonged later to 14th May 2016)
Results:
We aim to publish summary data from this survey.
Contact Information:
If you have any questions or difficulties with the survey, please contact Dr. Katja-Nicole Adamik, small animal clinic, Vetsuisse Faculty, University of Bern, Switzerland (katja.adamik@vetsuisse.unibe.ch)

Thank you for your time in completing this survey - we appreciate it!

Data S3

Contacted organizations (alphabetical order):

- American College of Veterinary Anesthesia and Analgesia (LISTSERV)
- American College of Veterinary Emergency and Critical Care diplomates (via LISTSERV)
- American College of Veterinary Emergency and Critical Care resident discussion board
- American College of Veterinary Internal Medicine (via LISTSERV)
- American College of Veterinary Surgery diplomates
- Bulgarian Association of Small Animal Veterinarians
- European university heads (Austria, Switzerland, Germany, France, Italy, Bulgaria, Poland, Sweden, Netherlands)
- European Veterinary Emergency and Critical Care Society members
- European College of Veterinary Internal Medicine diplomates
- European College of Veterinary Neurology diplomates
- European College of Veterinary Surgery diplomates
- European School for Advanced Veterinary Studies (website)
- Hellenic Veterinary Medical Society
- Latin American Veterinary Emergency and Critical Care Society
- Romanian Veterinary Emergency and Critical Care Society
- Spanish Small Animal Veterinary Association (website)
- Veterinary Emergency and Critical Care Society members
- VetsNow (discussion forum)

Data S4

Survey Part 1. Questions for the general part (Part 1) of the survey and answers from all 1134 respondents completing at least this part of the survey:

1. I work in:

	N	%
○ *Private practice*	489	43.1%
○ *University hospital*	319	28.1%
○ *Specialty practice/other*	326	28.7%

2. The total number of veterinarians working in my clinic/practice is:

	N	%
○ *1-10*	360	31.7%
○ *11-20*	262	23.1%
○ *21-30*	127	11.2%
○ *31-40*	101	8.9%
○ *>40*	284	25.0%

3. The cases I see are:

	N	%
○ *Mostly (>80%) referral cases*	415	36.6%
○ *Mostly (>80%) primary care cases*	187	16.5%
○ *A mixture of referral and primary care cases*	532	46.9%

4. I am a:

	N	%
○ *Intern*	28	2.5%
○ *Resident*	95	8.4%
○ *Diplomate*	435	38.4%
○ *DVM*	525	46.3%
○ *Other*	51	4.5%

5. My board program is:
(this question was only given to those answering “Resident” in question 4)

	N	%
○ *ACVAA*	10	10.2%
○ *ACVECC*	51	52.0%
○ *ACVIM*	2	2.0%
○ *ACVS*	4	4.1%
○ *ECVAA*	17	17.3%
○ *ECVECC*	1	1.0%
○ *ECVIM*	9	9.2%
○ *ECVN*	2	2.0%
○ *ECVS*	1	1.0%
○ *Other*	1	1.0%

6. My board certification is:
(this question was only given to those answering “Diplomate” in question 4)

	N	%
○ *ACVAA*	56	11.5%
○ *ACVECC*	200	41.2%
○ *ACVIM*	50	10.3%
○ *ACVS*	47	9.7%
○ *ECVAA*	28	5.8%
○ *ECVECC*	16	3.3%
○ *ECVIM*	40	8.2%
○ *ECVN*	6	1.2%
○ *ECVS*	28	5.8%
○ *Other*	15	3.1%

7. I am:

	N	%
○ *Male*	394	34.7%
○ *Female*	740	65.3%

8. I have practiced veterinary medicine for:

	N	%
○ *0-5 years*	237	20.9%
○ *6-10 years*	322	28.4%
○ *11-15 years*	233	20.5%
○ *16-20 years*	126	11.1%
○ *>20 years*	216	19.0%

9. The country in which I practice is:

	N	%
○ *Afghanistan*	1	0.1%
○ *Argentina*	2	0.2%
○ *Australia*	48	4.2%
○ *Austria*	13	1.1%
○ *Belgium*	10	0.9%
○ *Belize*	1	0.1%
○ *Brazil*	3	0.3%
○ *Bulgaria*	3	0.3%
○ *Canada*	48	4.2%
○ *Cayman Islands*	1	0.1%
○ *Chile*	3	0.3%
○ *China*	1	0.1%
○ *Colombia*	1	0.1%
○ *Cyprus*	1	0.1%
○ *Czech Republic*	1	0.1%
○ *Finland*	1	0.1%
○ *France*	25	2.2%
○ *Germany*	14	1.2%
○ *Greece*	13	1.1%
○ *Grenada*	2	0.2%
○ *India*	3	0.3%
○ *Ireland*	4	0.4%
○ *Israel*	7	0.6%
○ *Italy*	24	2.1%
○ *Japan*	2	0.2%
○ *Netherlands*	6	0.5%

- *New Zealand* 6 0.5%
- *Norway* 4 0.4%
- *Peru* 1 0.1%
- *Poland* 5 0.4%
- *Portugal* 15 1.3%
- *Romania* 4 0.4%
- *Russia* 2 0.2%
- *Slovakia* 1 0.1%
- *South Africa* 2 0.2%
- *South Korea* 2 0.2%
- *Spain* 83 7.3%
- *Sweden* 8 0.7%
- *Switzerland* 59 5.2%
- *Thailand* 1 0.1%
- *UK* 93 8.2%
- *USA* 610 53.8%

10. I use the following solutions for fluid resuscitation/shock (select one answer per line):

	Never (N)	*Rarely (N)*	*Sometimes (N)*	*Often (N)*
Isotonic crystalloids	2	9	19	1104
Hypertonic saline	168	389	442	135
Hydroxyethyl starch	138	232	498	266
Gelatin	1010	72	38	14
Dextran	985	92	47	10
Albumin	837	232	60	5
Plasma	425	428	249	32

11. I use the following solutions for fluid resuscitation/shock in the following conditions (select all that apply):

	Isotonic crystalloids (N)	*Hypertonic saline (N)*	*Hydroxyethy l starch (N)*	*Gelatin(N)*	*Dextran (N)*	*Albumin (N)*	*Plasma (N)*	*Other/ not applicable (N)*
Sepsis/SIRS	1101	264	188	524	31	28	613	23
Internal hemorrhage	1032	345	46	424	24	45	612	199
Head trauma	842	863	6	15	13	13	235	89
Gastric dilatation volvulus	1098	354	21	132	24	37	537	26
Gastrointestinal fluid losses	1109	126	113	205	27	26	502	23
Severe lung disease	958	170	22	82	11	11	264	103

12. I use the following solutions for general colloid osmotic pressure support (select one answer per line):

	Never (N)	*Rarely (N)*	*Sometimes (N)*	*Often (N)*
Hydroxyethyl starch	116	170	328	520
Gelatin	1014	54	45	21
Dextran	992	75	47	20
Albumin	566	331	188	49
Plasma	260	351	400	123

13. I use the following solutions for colloid osmotic pressure support in the following conditions (select all that apply):

	Hydroxyethyl starch (N)	*Gelatin (N)*	*Dextran (N)*	*Albumin (N)*	*Plasma (N)*	*Other/ not applicable (N)*
Sepsis/SIRS	779	50	41	332	665	106
Protein-losing nephropathy	580	39	28	279	382	262
Protein-losing enteropathy	747	50	34	335	443	174
Hepatic failure	579	30	35	278	630	212

14. I use the following albumin preparation(s) (select all that apply):

	N	%
○ *Human serum albumin 5%*	262	20.3%
○ *Human serum albumin 20% or 25%*	321	24.9%
○ *Lyophilized canine albumin*	182	14.1%
○ *Other*	19	1.5%
○ *I don't use any*	505	39.2%

15. My practice has a general policy/guideline on the use of colloids:

	N	%
○ *Agree*	304	26.8%
○ *Disagree*	726	64.0%
○ *I don't know*	104	9.2%

16. The synthetic colloid I most frequently use is:

	N	%
○ *Hydroxyethyl starch*	958	84.5%
○ *Gelatin*	49	4.3%
○ *Dextran*	31	2.7%
○ *I no longer use any*	71	6.3%
○ *I have never used any*	25	2.2%

17. The synthetic colloid I most frequently used was:
(this question was only given to those answering “I no longer use any” in question 16)

	N	%
○ *Hydroxyethyl starch*	65	91.5%
○ *Gelatin*	2	2.8%
○ *Dextran*	4	5.6%

Data S5

Frequency chart showing the relative frequency with which the 1134 survey respondents from different geographic regions use albumin products.

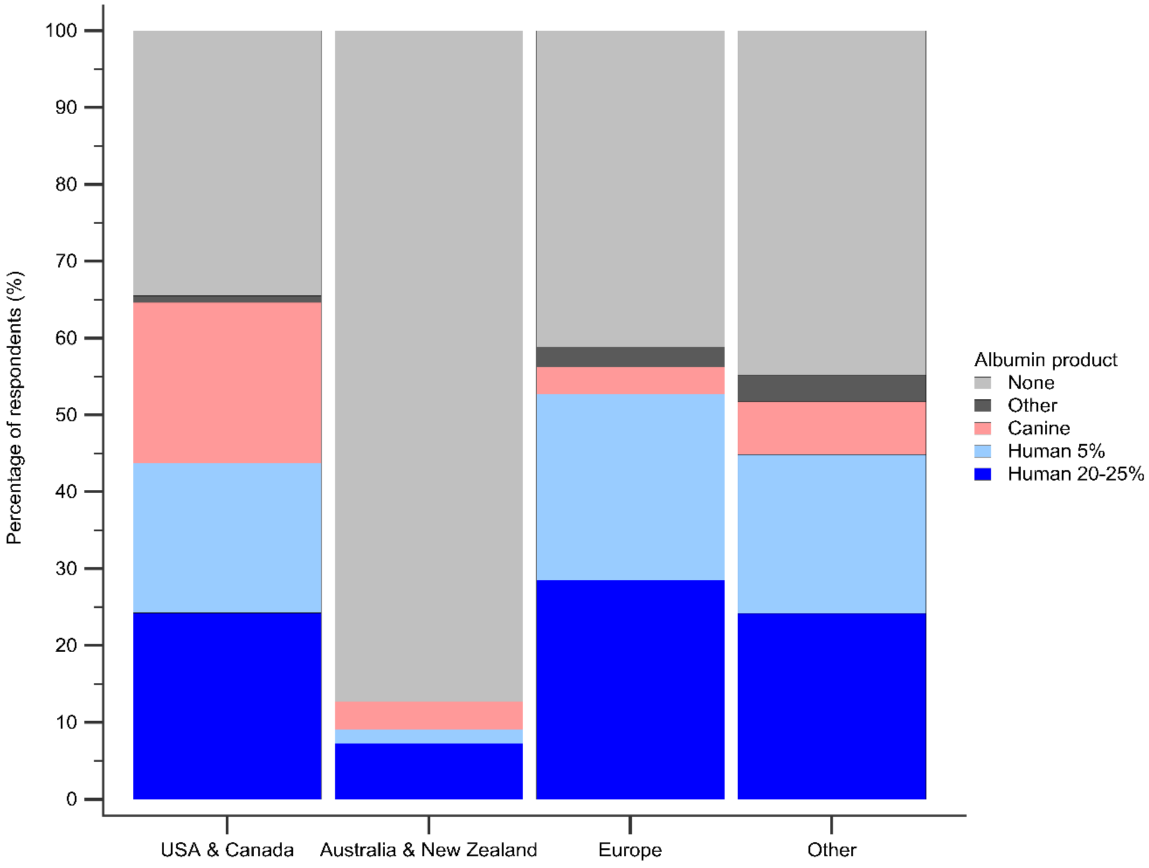

Data S6

Frequency chart showing the relative frequency with which the 1134 survey respondents with different qualifications (A) and from different geographic areas (B) agreed that a general policy for the use of colloids existed in their place of work.

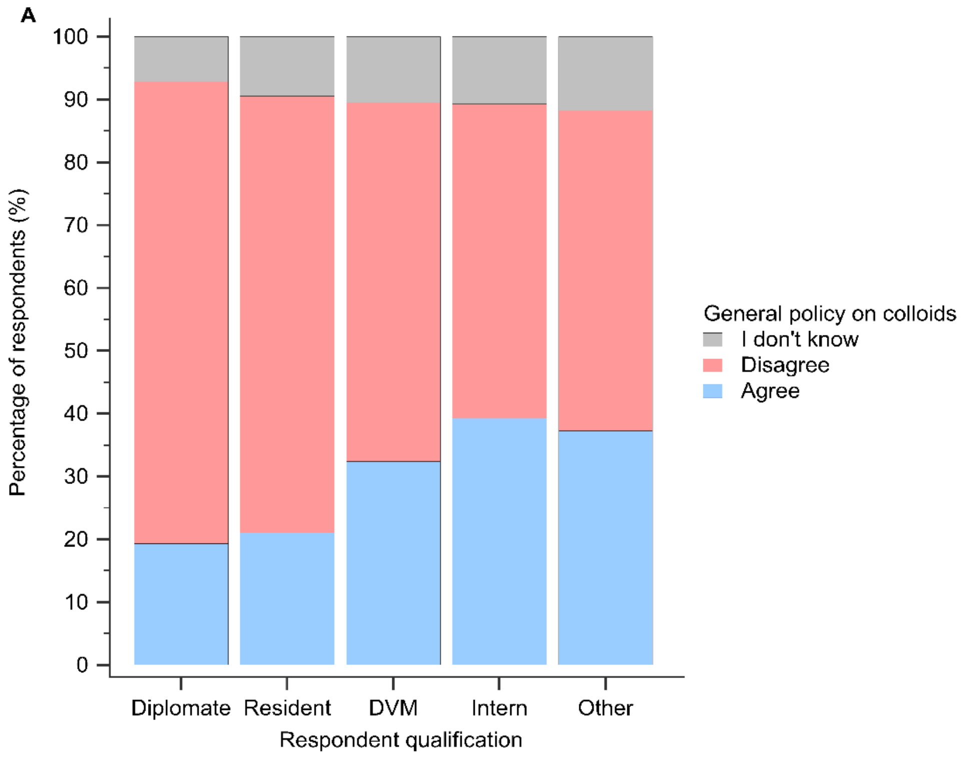

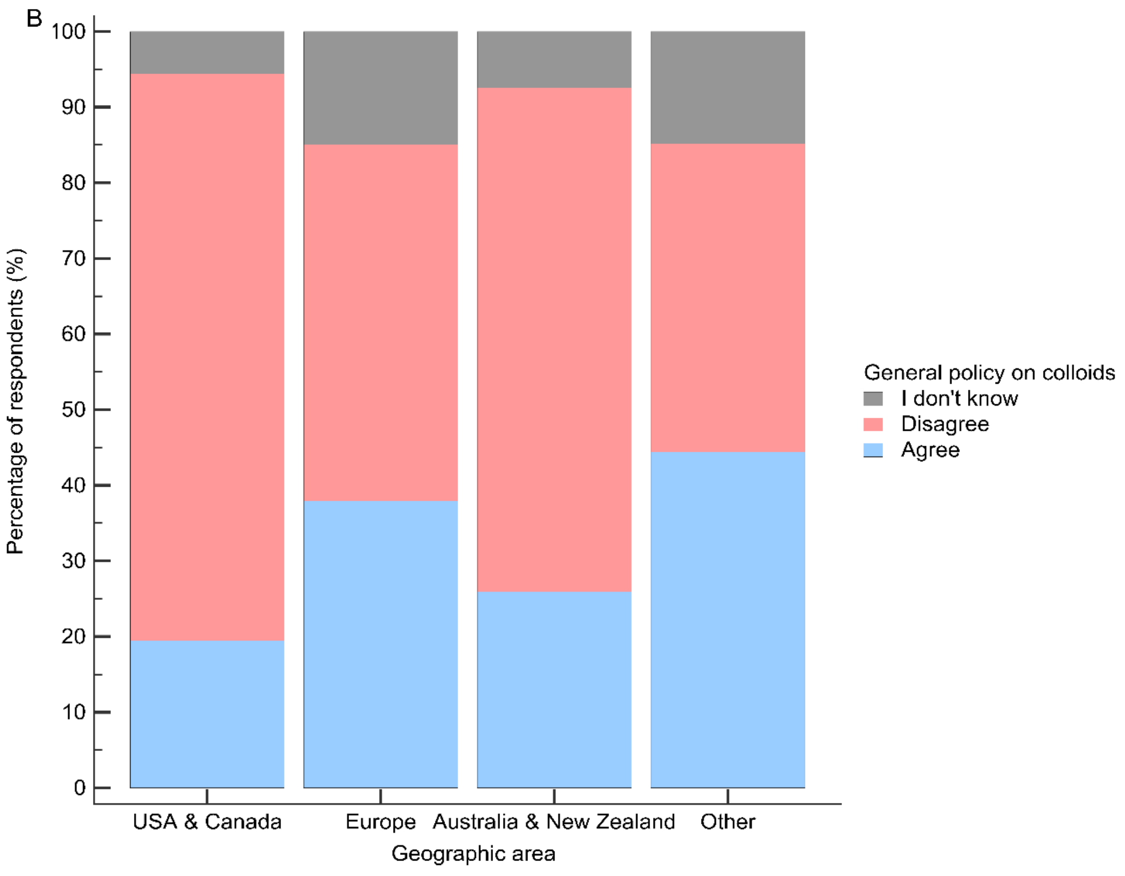

Data S7

Table with the most frequent synthetic colloid preparations used in different geographic regions reported by survey respondents.

Artificial colloid	Product	Respondents (N)					
		Australia & New Zealand	Europe	Other	USA & Canada	All	All (%)
Hydroxyethyl starch	6% HES 130/0.4	38	156	11	370	575	56%
	6% HES 130/0.42	0	25	1	4	30	3%
	10% HES 200/0.5	0	21	0	0	21	2%
	6% HES 200/0.5	0	47	1	6	54	5%
	6% HES 450/0.7	0	2	0	27	29	3%
	6% HES 650/0.75	3	17	5	184	209	20%
	Other HES	0	8	1	16	29	3%
Gelatin	3–4% succinylated gelatin	3	36	3	0	42	4%
	Other gelatin	0	5	1	0	6	1%
Dextran	Dextran	3	23	0	3	29	3%

Specific product types with ≤10 respondents are listed as “other”.

Data S8

Maximum daily doses for HES preparations in dogs and cats

**DOGS**	**HES product**								
Max daily dose	10% HES 200/0.5	10% HES 250/0.5	6% HES 130/0.4	6% HES 130/0.42	6% HES 200/0.5	6% HES 450/0.7	6% HES 670/0.75	Other	
0-10	0	1	23	2	6	1	23	3	59 (6.4%)
11-20	12	3	259	13	28	17	117	11	460 (49.6%)
21-30	3	0	156	5	8	4	38	3	217 (23.4%)
31-40	1	0	47	1	3	4	13	1	70 (7.6%)
41-50	1	0	53	4	2	2	2	1	65 (7.0%)
>50	0	0	5	0	0	0	1	0	6 (0.6%)
I don't know/ not applicable	2	0	26	4	3	1	9	5	50 (5.4%)

**CATS**	**HES product**								
Max daily dose	10% HES 200/0.5	10% HES 250/0.5	6% HES 130/0.4	6% HES 130/0.42	6% HES 200/0.5	6% HES 450/0.7	6% HES 670/0.75	Other	
0-10	5	2	148	9	18	13	68	6	269 (29.0%)
11-20	9	2	268	10	20	13	92	10	424 (45.7%)
21-30	1	0	91	2	3	1	16	1	115 (12.4%)
31-40	1	0	10	0	3	0	4	0	18 (1.9%)
41-50	0	0	17	3	2	0	0	1	23 (2.5%)
>50	0	0	3	0	0	0	0	0	3 (0.3%)
I don't know/ not applicable	3	0	32	5	4	2	23	6	75 (8.1%)

Data S9

Frequency chart showing observed adverse reactions to AC use reported by 927 hydroxyethyl starch users (A), 49 gelatin users (B) and 25 dextran users (C) responding to the survey.

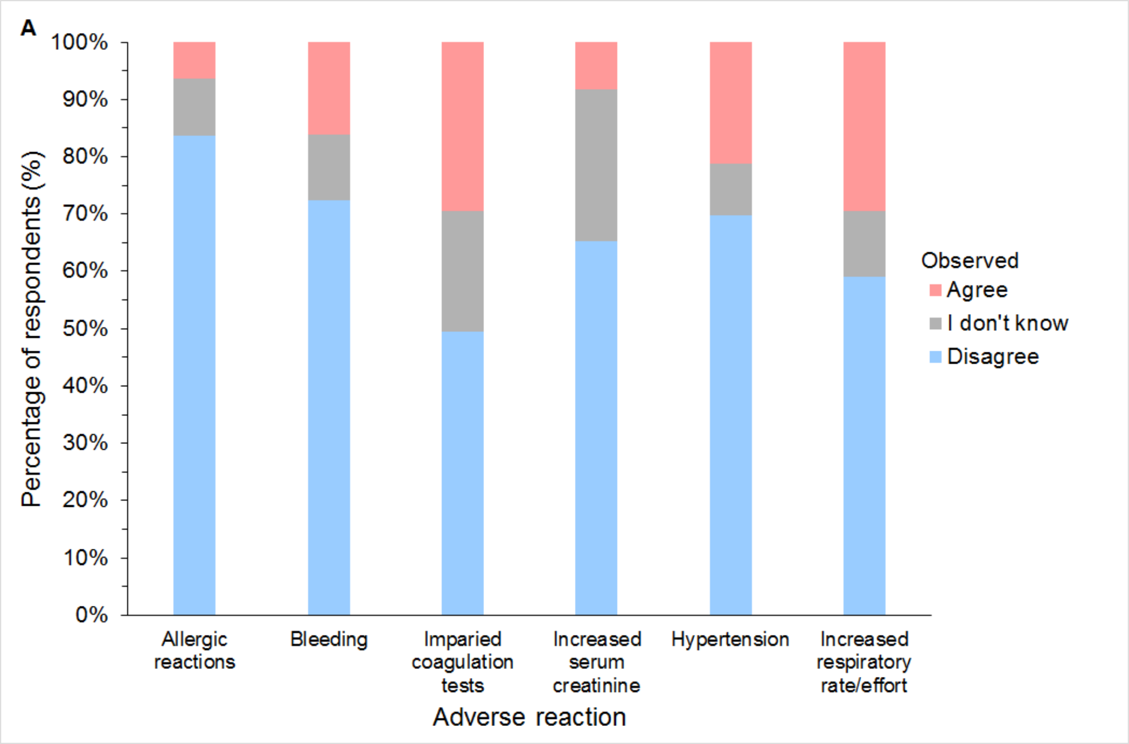

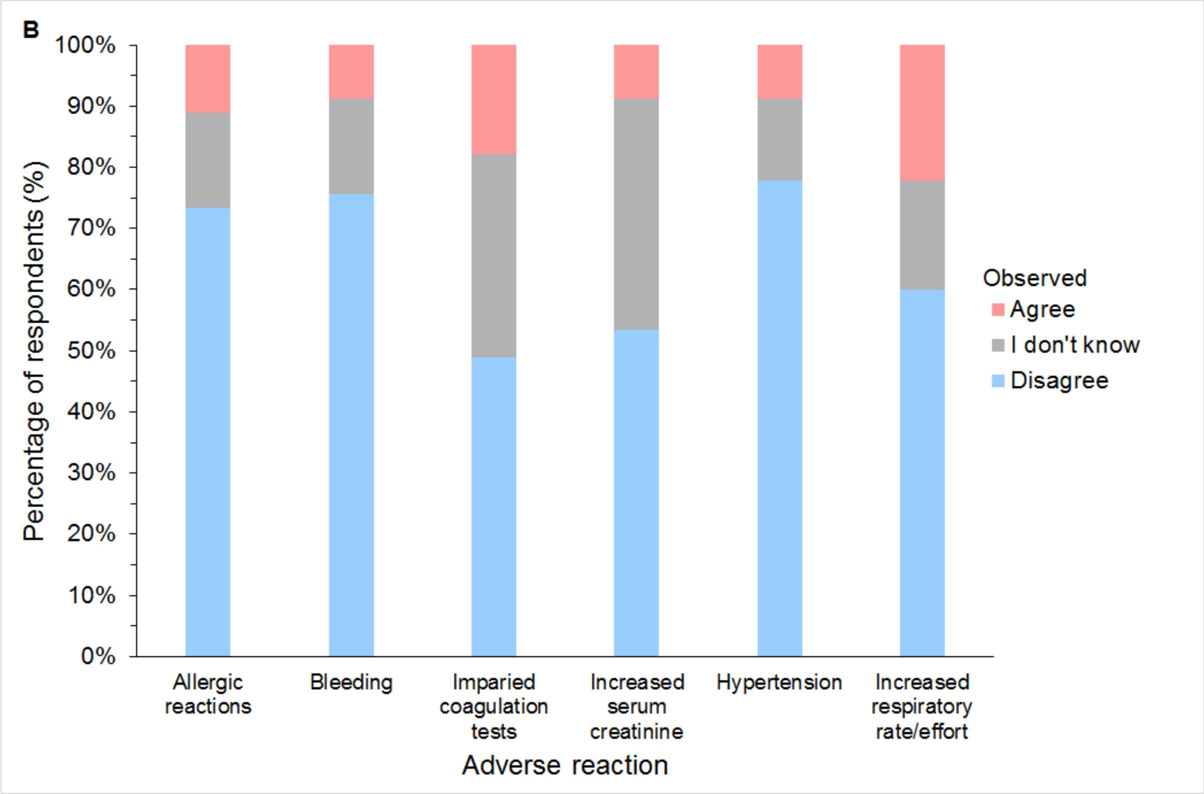

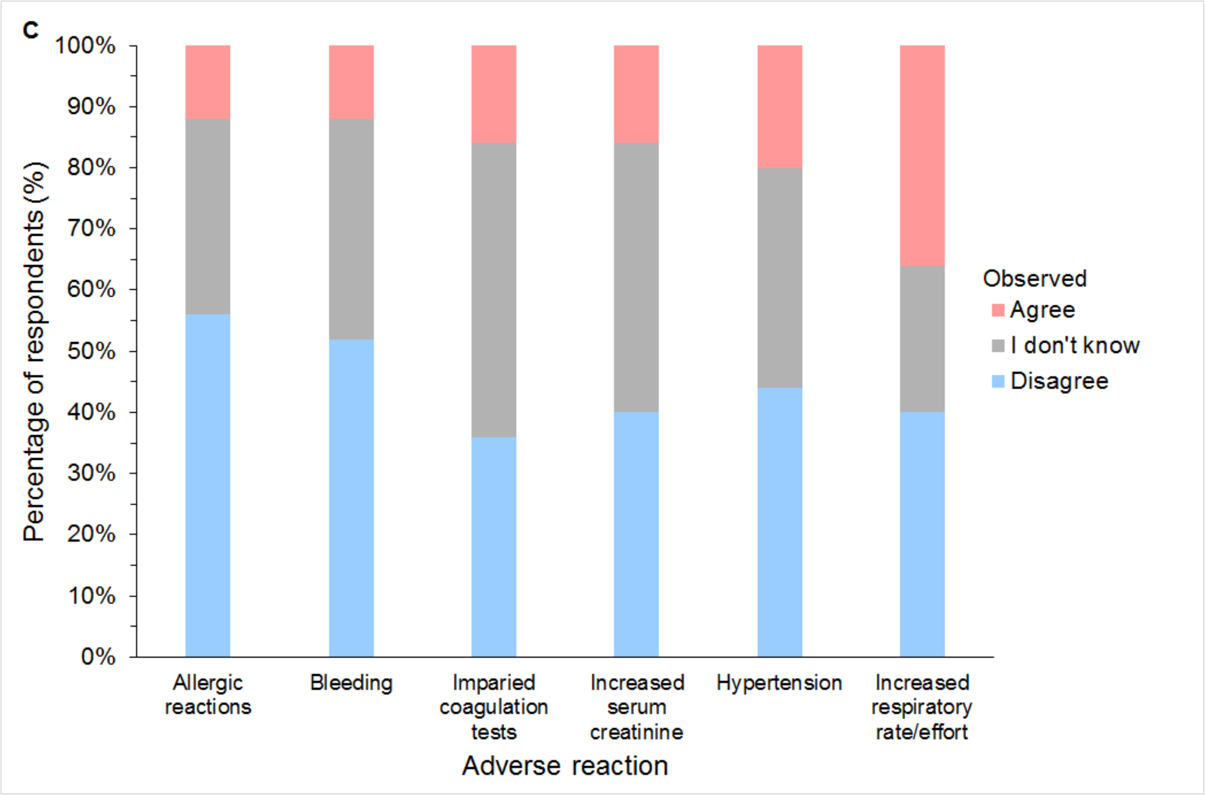
C
100%
90%
80%
70%
60%
50%
40%
30%
20%
10%
0%
Percentage of respondents (%)
Allergic reactions
Bleeding
Imparied coagulation tests
Increased serum creatinine
Hypertension
Increased respiratory rate/effort
Adverse reaction
Observed
Agree
I don't know
Disagree
